# Characterizing the role of human behavior in the effectiveness of contact-tracing applications

**DOI:** 10.3389/fpubh.2023.1266989

**Published:** 2023-11-03

**Authors:** Ariadna Fosch, Alberto Aleta, Yamir Moreno

**Affiliations:** ^1^Institute for Biocomputation and Physics of Complex Systems, University of Zaragoza, Zaragoza, Spain; ^2^CENTAI Institute, Turin, Italy; ^3^Department of Theoretical Physics, University of Zaragoza, Zaragoza, Spain

**Keywords:** digital contact-tracing, contact-tracing apps, COVID-19, epidemic modeling, human behavior, multilayer networks, computational modeling

## Abstract

**Introduction:**

Although numerous countries relied on contact-tracing (CT) applications as an epidemic control measure against the COVID-19 pandemic, the debate around their effectiveness is still open. Most studies indicate that very high levels of adoption are required to stop disease progression, placing the main interest of policymakers in promoting app adherence. However, other factors of human behavior, like delays in adherence or heterogeneous compliance, are often disregarded.

**Methods:**

To characterize the impact of human behavior on the effectiveness of CT apps we propose a multilayer network model reflecting the co-evolution of an epidemic outbreak and the app adoption dynamics over a synthetic population generated from survey data. The model was initialized to produce epidemic outbreaks resembling the first wave of the COVID-19 pandemic and was used to explore the impact of different changes in behavioral features in peak incidence and maximal prevalence.

**Results:**

The results corroborate the relevance of the number of users for the effectiveness of CT apps but also highlight the need for early adoption and, at least, moderate levels of compliance, which are factors often not considered by most policymakers.

**Discussion:**

The insight obtained was used to identify a bottleneck in the implementation of several apps, such as the Spanish CT app, where we hypothesize that a simplification of the reporting system could result in increased effectiveness through a rise in the levels of compliance.

## 1. Introduction

Contact tracing (CT) is one of the most effective epidemic control strategies, as it allows cutting the disease transmission chains by isolating potentially infected individuals before they can further spread the pathogen (1). However, during the early days of the COVID-19 pandemic, this classical strategy was hardly effective due to the long turnaround of tests, and the presence of presymptomatic ineffectiveness and mild symptomatology in a large proportion of cases. As a consequence, many countries promoted the use of novel digital contact tracing (DCT) strategies, based on the use of smartphone apps. These apps, also known as CT apps, rely on several technologies to register interactions between individuals and warn those who recently had contact with someone who turns out to be infected. This way, these individuals could quarantine themselves preventively, much sooner than with classical contact tracing (2).

These apparent benefits produced an explosive growth of CT apps even though there was no empirical evidence of the effectiveness of these tools (3). In less than a year, researchers from MIT identified at least 80 different CT apps deployed over 50 countries (4). Concurrently, many modeling studies tried to understand whether these apps could be effective, and it was generally accepted that the adoption rate should be around 60% of the population for pandemic mitigation (2, 5–7). Nonetheless, current empirical evidence signals that in practice most apps were not as effective as expected and that many models were too optimistic (8).

For example, health authorities in New South Wales, Australia, compared the performance of the Australian CT app vs. conventional contact tracing during an outbreak of 619 cases (9). They observed that only 137 of the infectees had the app, which corresponds to an adoption rate of 33%. Among these 137 users, only 32 warned at least one contact using it. In total, there were 79 contacts notified, or roughly 3 per index case. In contrast, conventional contact tracing revealed 25,300 close contacts, 40 per index case. Moreover, the app only detected 17 (<0.1%) new contacts not identified through conventional tracing. Based on these outcomes, Australian health workers considered that the app was not useful for them and that its use actually increased their burden (9).

Similar conclusions were also reached in other countries. In Finland, out of 4,557 PCR-positive cases, only 541 warned their contacts (12%). Besides, most people that received the warning had already been alerted through traditional contact tracing, since the procedure to notify contacts with the app was rather slow. In total, only 8 (0.3%) people reported having changed their behavior due to the app notification (10). In Switzerland, with an adoption rate of 26%, only between 20 to 40% of the users triggered an exposure notification upon receiving a positive test (11). In Belgium, the adoption rate was 28%, and only 43% of the users employed it to notify their contacts (12). The generalized low adoption levels may have played a role in limiting the effectiveness of DCT strategies. However, the level of reporting among users was also strikingly low across these countries, which could greatly hinder the efficacy of the procedure even if large levels of adoption were to be achieved.

Though most countries categorized CT apps as ineffective interventions, a study about the British CT app concluded otherwise. Even when it only had an adoption rate of 29%, they estimated that during an outbreak of over half a million individuals, the CT app resulted in 1.7 million notifications sent to potential contacts, roughly 3 per case. With an estimated secondary attack rate of 6%, and assuming that 65% of individuals adhered to quarantine, it was estimated that between 4,200 to 8,700 deaths were averted by the app, claiming that it was highly successful (13).

Interestingly, both the percentage of adoption and the number of contacts alerted per index case were very similar to the ones found in the Australian study. Therefore, the apparent success of the intervention probably derives from the difference in the size of both outbreaks rather than from the good performance of the app. While the outbreak in Australia was relatively small and conventional contact tracing was extraordinarily effective, identifying 40 close contacts per index case, the outbreak in the UK was much larger, and manual contact tracing only identified 2 close contacts on average (14).

Overall, it is clear that the worldwide performance of DCT systems was far from ideal. But was this solely derived from the low penetration of the app or other aspects of human behavior may have influenced their performance? From the empirical implementations, we identified that compliance with reporting is an important factor to consider. Moreover, it is also likely that the reluctance of the population toward app adoption may have induced unexpected temporal delays between the start of the CT app campaign and its mass adoption, which could have also hindered their performance.

Now that the pandemic phase of COVID-19 has come to an end, it is time to gather all evidence available to generate more complete CT app models that can better inform policy-making. To this end, we propose a hybrid model that combines epidemic and human behavior dynamics to describe the co-evolution of a disease outbreak and the CT app adoption process over a single population. Through its use, we assessed the impact of three human behavior parameters (maximal app adoption, average reluctance toward adoption, and reporting compliance) on the effectiveness of CT apps. This allowed us to extract some general recommendations for healthcare authorities and policy-makers, which can be interpreted as a set of good practices for future implementations of DCT systems.

## 2. Methodology

### 2.1. Population structure

Communicable diseases spread through the interaction between an infected individual and a susceptible (or healthy) one. To model these interaction patterns, epidemic models often rely on networks. These mathematical representations are composed of a set of entities (also known as nodes) and the pairwise interactions between them (known as edges or links) (15).

**Figure 1:**
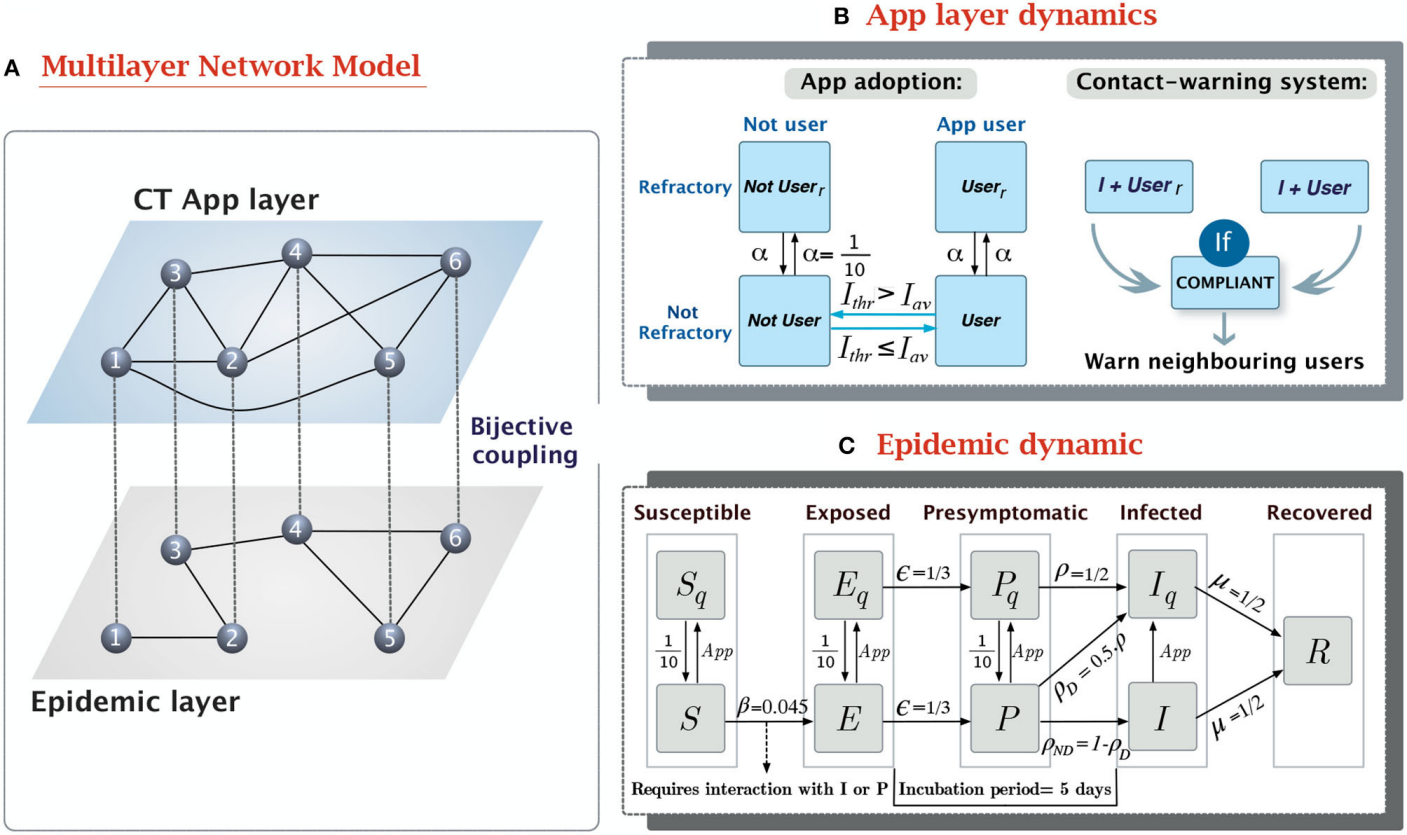
Overview of the epidemic-CT app multilayer network model. **(A)** Schema of the multilayer network structure and the coupling between layers. The links connecting nodes in different layers are a visual aid to reflect that both nodes represent the same individual in two contexts. **(B)** Compartmental model of the dynamic in the CT app layer. It contains two separate dynamics, the app adoption dynamic, and the app effect. **(C)** Compartmental model for the epidemic layer. It is based on a modified SEPIR epidemic model (17), with the addition of a presymptomatic state (*P*) and quarantined versions of the *S*, *E*, *P*, and *I* states. The transition rates have been defined to produce outbreaks reassembling the 1st wave of the COVID-19 pandemic.

In our model, we have considered two different types of interaction patterns between the same set of 10,000 individuals. The first connectivity pattern reflects the in-person interactions. This is the population structure in which the epidemic dynamic spreads. Meanwhile, the second connectivity pattern reflects the Bluetooth interactions registered by the CT app, which are only a proxy of the real in-person contacts. Both patterns represent connections between the same set of nodes through different edge distributions. Therefore, they can be represented as a multilayer network with two layers (16), the epidemic layer and the CT app layer. See Figure 1A for a schematic representation of the multilayer network structure.

The connectivity pattern of the epidemic layer was defined to follow the contact distribution observed in survey data from the Italian population (18, 19). In graph theory, the distribution of the number of contacts per individual is often called the degree distribution, and its shape and average value define many of the topological properties of a network (15). Prior research has indicated that the degree distribution of in-person contact networks follows a negative binomial shape (18), and thus it can be completely characterized through its average value and dispersion parameter. To estimate them from survey data we followed the approach described in Lu et al. (20). We first estimated the average number of interactions per individual (average degree 〈*k*〉) from the age-mixing matrices in Mistry et al. (19) (〈*k*〉 = 11.92), and then we obtained the dispersion parameter (*r*) by fitting a negative binomial distribution to the age-aggregated data from the POLYMOD study (*r* = 2.426) (18).

To define the connectivity pattern of the CT app layer we just expanded the in-person contact network with random edges. These spurious links reflect interactions meeting the inclusion criteria of the CT app (location and duration) but not entailing a risk of infection, because of other protective measures or physical barriers between the users. For instance, in Australia, up to 61% of the contacts identified by the app were workers in adjacent rooms, customers in neighboring restaurants or even people waiting in separate cars at COVID-19 drive-through testing clinics (9). Thus, we duplicated the number of contacts associated with random encounters in this network. Given that random contacts encompass approximately 25% of the 11.92 average daily interactions of a person (19), this yields an average degree in the CT app network of 〈*k*〉_App_ = 14.81.

To evaluate the sensitivity of our results to the population structure characteristics, we repeated the analysis for populations with two non-empiric degree distributions [Erdős and Rényi random graph (21) and a Scale-Free network (15)] and a very similar 〈*k*〉. More details about the network definition and the fitting process are available in Supplementary Methods.

### 2.2. Epidemic model

The dynamics of epidemic contagion have traditionally been modeled through compartmental models. This approach assumes that individuals can be classified in one of the states (compartments) of the model and they can change their state depending on the probability of transition between compartments (15).

We described the natural history of SARS-CoV-2 using the compartmental model shown in Figure 1C. This model is based on the Susceptible-Exposed-Presymptomatic-Infected-Removed (SEPIR) model (17), which has already been used to model SARS-CoV-2 progression (22). In short, the SEPIR model assumes susceptible individuals (*S*) can be infected by interacting with their neighbors with infectious capabilities, presymptomatic (*P*) or infected (*I*). After contagion, *S* individuals transition toward the exposed state (*E*), in which they are already infected but not yet infectious. After this latent phase, infected individuals gain contagion power, despite still not showing symptoms, this is what is known as the presymptomatic infected state (*P*). As symptoms appear *P* individuals transition toward the symptomatic infectious state (*I*), in which they will remain until they lose contagiousness, thus transitioning to the removed state (*R*).

This basic epidemic model was modified to incorporate quarantined equivalents to the *S*, *E*, *P*, and *I* states (*S*_*q*_, *E*_*q*_, *P*_*q*_, and *I*_*q*_). In them, disease follows the same progression as in their non-quarantined counterpart, but *P*_*q*_, and *I*_*q*_ cannot infect others and *S*_*q*_ cannot become infected. Transitioning from a free state to its quarantined analog occurs mainly through the CT app's warning system. All individuals who receive a message from the app are forced to enter a 10-day preventive quarantine regardless of their current state. Additionally, a fraction of the daily new symptomatic individuals (*P*→*I* transitions) are detected in healthcare testing and quarantined 1 day after developing symptoms. By limiting healthcare detection to symptomatic cases on their first day of symptoms we reflect the large fraction of individuals with mild or asymptomatic COVID-19 manifestations, who would never be detected without additional control strategies. Contrary to the preventive quarantines, confirmed positive cases (*I*_*q*_) remain under quarantine until their complete loss of contagiousness.

The transition rates across compartments were defined based on epidemiological metrics empirically estimated during the Alpha variant of COVID-19, like the mean incubation period (IP), the generation time, or the basic reproductive number (*R*_0_). More specific definitions of these epidemiological metrics can be found in Supplementary Table 1.

The IP defines the average time between infection and the start of symptoms onset. In the case of the Alpha variant of COVID-19, several studies reported it was around 5 days, e.g., 4.9 days (95% credible intervals, CrI, 4.4–5.4) (23) or 5.1 days (95% CI, 4.5–5.8 days) (24). Another relevant epidemic parameter is the mean generation time, which indicates the average delay between two lineages of a generation (25). In the case of the Alpha variant, it was estimated to be 7.12 days (95% CrI 6.27–8.44) (23). Based on these parameters, we assumed the transition rates between *E*→*P* and *P*→*I* to be ϵ = 1/3 and ρ = 1/2 respectively, resulting in an average incubation period of 5 days. To reflect the 7-day mean generation time, the transition rate from *I*→*R* (removal probability) was assumed to be μ = 1/2. Note that in this case, the μ does not represent the probability of complete recovery from the symptoms of COVID-19; it merely reflects the loss of contagious capacity of an infectee, either due to natural causes or due to behavioral changes (contact reduction).

The last parameter of the epidemic compartmental model is the transmissibility parameter (β). This parameter reflects the infection rate of the disease and we defined it to reflect the *R*_0_ observed in European countries during the 1st wave of the COVID-19 pandemic (2 ≤ *R*_0_ ≤ 3) (26).

**Figure 2:**
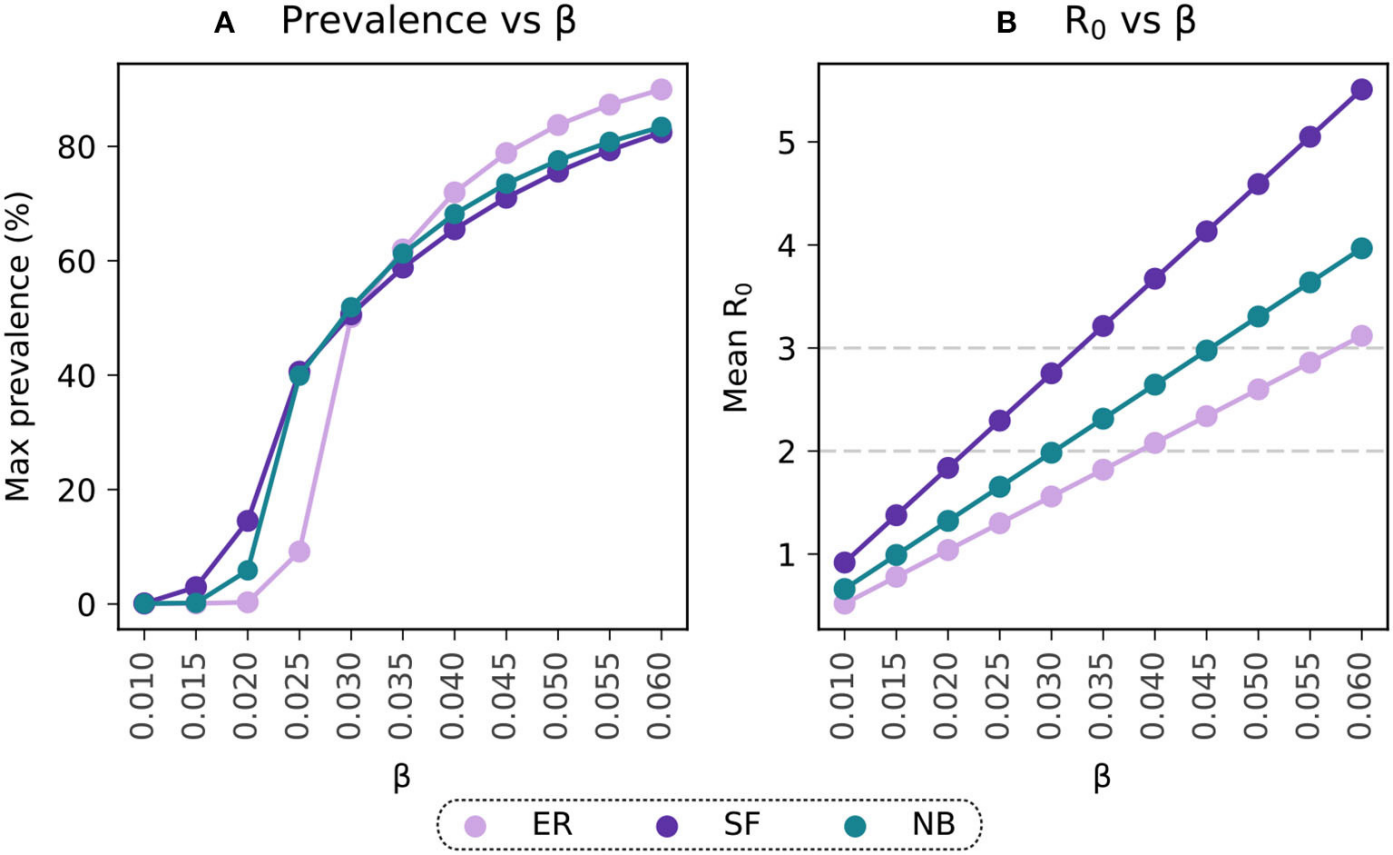
Selection of the transmissibility parameter on the basis of *R*_0_. **(A)** Maximal prevalence for simulations with ranging values of β. **(B)** Basic reproductive number estimated for simulations with varying β. Light-violet trends represent the results for the Erdo˝ s-Rényi connectivity matrix (ER), purple trends reflect estimations for the Scale-Free (SF) population and the blue points reflect the results for the Negative Binomial distribution (NB) fitted to data from contact patterns surveys.

*R*_0_ is an epidemiological metric widely used to estimate the difficulty of controlling epidemic progression. For *R*_0_ < 1 an epidemic outbreak naturally dies out, as the number of recovered individuals will exceed the number of new infections. Meanwhile, *R*_0_>1 indicates that the pathogen continues spreading (15). For a pathogen with presymptomatic infectivity, the relationship between β and *R*_0_ is given by


(1) $$\begin{array}{l} R_{0}=\beta \left(\frac{1}{\rho }+\frac{1}{\mu }\right){\text{λ}}_{\mathbb{R}}\left(A_{ij}\right) \end{array}$$

where β, ρ, and μ represent the epidemic parameters, and λ_ℝ_ is the real component of the largest eigenvalue of the network's adjacency matrix (27). Hence, for a given β, the actual value of *R*_0_ will depend on the network under consideration. We calibrated the model to ensure it resulted on an *R*_0_ = 3 using our in-person contact network estimated from mobility data. This yields β = 0.045, see Figure 2B.

We also relied on empirical data to define the transition rates for healthcare detection and the duration of preventive quarantines. Seroprevalence studies have shown that during the first wave, only 10% of the cases were detected, increasing to 60–70% in subsequent waves (28). Thus, we assumed that half of the new daily symptomatic were detected on the first day of infection (δ = 0.5) and that preventive quarantines lasted for 10 days, as suggested in the COVID-19 quarantine protocol followed by the Spanish government during most of the pandemic (29).

The epidemic parameters described above were also used for the simulations evaluating the sensitivity of the results to the population structure. Given the dependence of *R*_0_ on the network structure, the *R*_0_ values from the Erdős-Rényi and Scale-Free simulations are different than the one in the negative binomial distribution. However, their maximal prevalence levels show similar values for the whole range of β under consideration, Figure 2A.

A different sensitivity analysis was also performed to assess the impact of the epidemic parameterization on the results obtained. In this case, we simulated outbreaks with a shorter incubation period (IP = 3.5 days), more similar to the Omicron variant (23), and different *R*_0_ values. More details about how this sensitivity analysis can be found in the Supplementary Results.

### 2.3. Contact-tracing app model

The dynamical model in the CT app layer reflects two different processes, the adoption of the CT app and the spread of the warning notifications to potentially infected individuals (see Figure 1B).

The app adoption dynamics describe the decision-making process of each individual to download or remove the CT app depending on their reluctance level and the pressure of external factors. Prior research has described binary decision-making in rational individuals (like adherence to riots or trends) using threshold dynamics. These models assume each individual in the population has an intrinsic reluctance threshold for adopting a specific behavior. This represents the minimum level of external influence (e.g., peer pressure or media information) required for an individual to adopt the behavior. The dynamical process then considers that an individual will adopt the behavior if the adoption pressure is higher than their reluctance level or otherwise they will remain in the non-adopting state (30).

Our model assumes that disease progression acts as a positive pressure toward app adoption, as it has been observed that fear of infection will push more reluctant individuals to overcome their concerns and download the app (31). Because of this, we defined the reluctancy threshold for each individual as the minimal level of infection (7-day incidence/100,000 inh.) triggering their adoption of the app. More intuitively, the app adoption dynamics assume that individuals will only use the CT app while they feel at risk (the incidence level is above their reluctancy threshold), and they will uninstall it once the incidence returns below threshold. Generally, the decision to download or remove the CT app is not made on a daily basis, even if the environmental conditions have changed. To incorporate this factor, we introduced a refractory period after the decision-making event equal to the duration of the preventive quarantine (10 days).

To model the heterogeneous reluctance levels between individuals we sampled their reluctancy threshold from a Poisson distribution with a pre-defined average reluctancy threshold (*I*_thr_). This distribution was modified in both extremes to include a fraction of individuals with extreme responses:

- Non-adopters: Individuals who would never adopt the app, either because they are too concerned about their privacy, or they do not have access to a compatible smartphone. In the case of European countries, prior studies have shown that at least 30% of the population may be unable to acquire CT apps (5). We used this value to set the upper bound in the maximal number of adopters to 70%.
- Early adopters: Pioneer individuals without reluctance toward app adoption. They download the app right after its implementation and only uninstall it after the complete extinction of the epidemic outbreak (*I*_*thr*_ = 0 cases/100, 000 inh.). We considered only 1% of the population to be early adopters.

The population's *I*_thr_ can be interpreted as the cultural differences between different populations, in terms of their willingness to adopt the CT app. Populations with a high average threshold require more time to reach the level of infection triggering the generalized adoption of the apps, while low threshold populations will adopt the CT app more easily.

The second dynamic implemented in the CT app layer is the reporting system. Only compliant app users who test positive for SARS-CoV-2 (individuals transitioning to the *I*_*q*_ state) will report their infection in the CT app and activate the contact warning system. This will cause all their Bluetooth contacts (neighbors in the app layer) who are also active users to enter a 10-day preventive quarantine. We assumed that compliance is only related to reporting, thus, individuals who are labeled as non-compliant will still quarantine themselves since they willingly downloaded the app. Individuals who do not report their status nor follow the app recommendations are those who do not have the app installed.

A list of the parameters used for the epidemic-CT app model is available in the Supplementary Table 2.

### 2.4. Simulations

We assessed the impact of the CT app intervention by measuring the difference in peak incidence and total prevalence between a simulation with the CT app and a baseline scenario without it. Both simulations were initialized with the same initial conditions and iterated for 500 time steps (500 days), enough time to observe a complete dynamic in both layers. To reduce the effects of stochastic noise, we repeated each simulation 1,000 times starting from different initial conditions.

Before estimating the effectiveness of the CT app, we removed all repetitions not resulting in an effective outbreak (max(*I*) < 1%) and aligned the surviving ones following kiss et al. (32) (see Supplementary Section 1.3 for a description of the alignment). The effectiveness of the CT app was measured over the average response of the surviving repetitions in each simulation and it was estimated by using the relative reduction in peak incidence (Δ_*i*_) and maximal prevalence (Δ_*p*_). For both cases Δ was defined as


(2) $$\begin{array}{l} \text{Δ}=1-\frac{\text{max}\left(I_{\text{CT app}}\right)}{\text{max}\left(I_{\text{Baseline}}\right)} \end{array}$$

where *I*_CT app_ is the value of the metric (peak incidence or maximal prevalence) in the scenario with an active CT app, and *I*_Baseline_ is the same metric for the scenario without the CT app.

The relative peak incidence reduction is associated with a flattening of the epidemic curve, indicating the potential of the CT app to reduce the speed of epidemic propagation and the pressure on the healthcare system. Meanwhile, maximal prevalence reduction is an indicator of the overall impact of the CT app in reducing the total number of infections, providing a more global perspective of the effect of the apps. The code to reproduce the analysis is available on the following public repository https://github.com/AFosch/Epi-CTapp.

## 3. Results

### 3.1. Scenario description

We evaluated the influence of three factors of human behavior in the effectiveness of CT apps: the maximal percentage of adoption, the average reluctancy toward app adoption and the fraction of compliant users. To this end, three hypothetical scenarios were implemented: the voluntary adoption scenario (where 100% compliance is assumed), the imposed adoption one (with zero reluctance toward app adoption) and the “adherence & compliance” scenario (only constraining the maximal level of adoption). These scenarios can also be interpreted according to the characteristics of the app adoption campaigns followed by different countries during the COVID-19 pandemic. In European countries, most countries relied on voluntary adoption campaigns, where adherence is not compulsory and it only depends on the population's willingness to download the app (its average reluctance threshold). In this scenario, complete compliance is assumed under the hypothesis that individuals who voluntarily decide to adhere to the strategy will also be more likely to comply with it. Contrarily, the imposed adoption strategy assumes that individuals are forced to download the app from the start of the epidemic outbreak (*I*_thr_ = 0 cases/100, 000 inh.) but no control is exhorted over their use.

**Figure 3:**
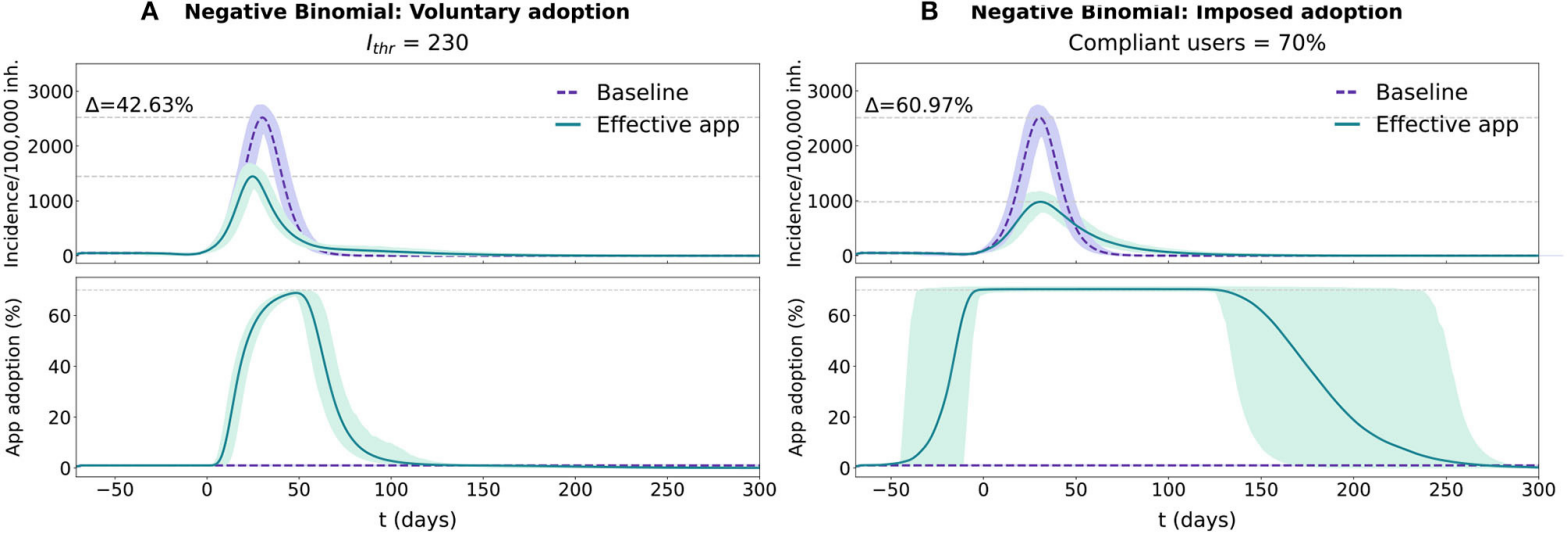
Temporal evolution for the epidemic and app adoption dynamics in the voluntary adoption and imposed adoption scenarios in a population with a realistic distribution (Negative Binomial fit with survey data). In each panel two conditions are tested, purple traces reflect the baseline condition (no app adoption) and the green ones reflect the effective CT app situation. The results for both conditions are represented with the average and 95% CI across 1,000 repetitions. Epidemic progression is reported using the 7-day incidence/100,000 inh. (more details available in Supplementary Section 1.4), while CT app adoption is reported with the percentage of the population using the CT app. **(A)** Simulation for the voluntary adoption scenario with an *I*_thr_ = 230 cases/100, 000 inh. **(B)** Simulation for the imposed adoption scenario with a fraction of compliant users = 70%.

The differences between these two approaches are more evident when comparing their evolution over the same baseline epidemic outbreak. In the voluntary adoption scenario (Figure 3A), app adoption only starts rapidly growing after the epidemic outbreak has reached the population's average reluctancy threshold (*I*_thr_ = 230 cases/100, 000 inh. in this case). As the epidemic progresses, the number of users continues to increase, reducing its adoption rate as the epidemic starts its decline. When the incidence level decreases below the threshold, the number of users rapidly declines, until the complete removal of the app around *t* = 120. In this scenario, the average reluctancy threshold controls the start of the adoption process and the time of removal of the app, conditioning the total duration of the control strategy. Lower reluctancy produces wider windows of app adoption, resulting in higher detection rates and increased app effectiveness. This can be observed in more detail in Supplementary Figure 3A.

In the imposed adoption scenario the app adoption dynamic differs (see Figure 3B). Since the average reluctancy threshold is defined to *I*_thr_ = 0 cases/100, 000 inh., app adoption rapidly grows at the start of the simulation, reaching the maximal level of adoption (70%) before the epidemic's exponential growth phase (*t* = −4). Maximal adoption is maintained for the rest of the epidemic outbreak and the app removal cascade will only start after the complete extinction of the disease. In this scenario, the maximal number of adopters is maintained for the whole duration of the epidemic, regardless of its size (see Supplementary Figure 3B). Thus, compliance affects the effectiveness of the strategy through a reduction in the performance of the reporting system, not through a change in the duration of the adoption window.

Finally, the “adherence & compliance” scenario relaxes the assumptions of complete compliance and no reluctance toward app adoption assumed respectively, in the voluntary and imposed adoption scenarios. It represents a more realistic scenario, where the only constraint introduced over the human behavior parameters is on the upper bound of the number of adopters (set to 70% of the total population). As mentioned in Section 2.3, the number of app users is intrinsically upper-bounded by the proportion of individuals who do not have an appropriate device (30% of the population).

### 3.2. Exploratory analysis of the human behavior parameters

**Figure 4:**
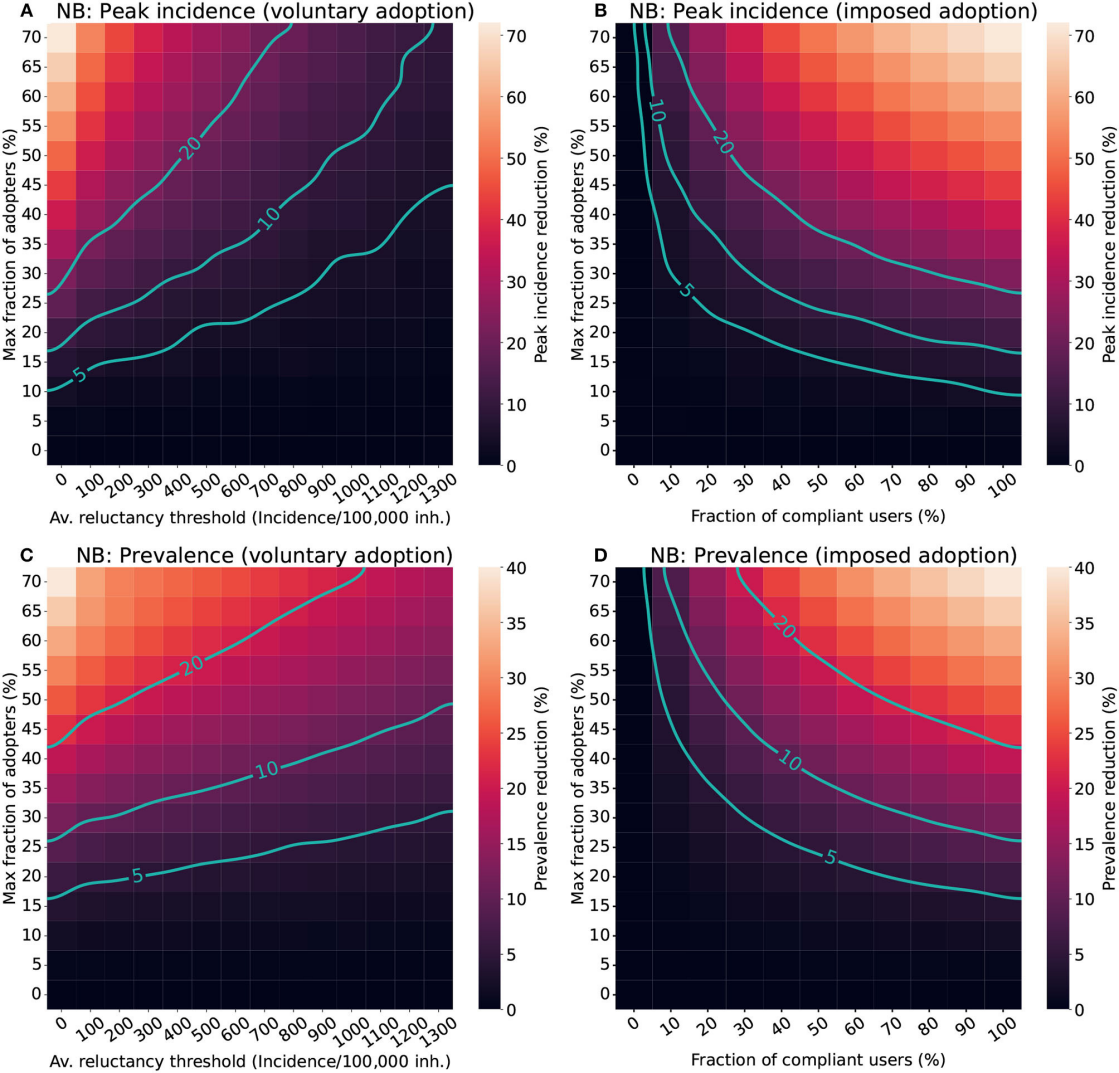
Impact of different factors of human behavior in the effectiveness of CT apps in a population with a Negative binomial distribution (NB). For the voluntary adoption scenario **(A, C)**, the parameters explored are the average reluctance threshold and the maximal fraction of adopters. Meanwhile, in the imposed adoption scenario **(B, D)**, changes in the fraction of cooperative users and the maximal fraction of adopters are explored instead. The color scale reflects the average reduction produced by the CT app (Δ) in the peak incidence (top panels) or maximal prevalence (lower panels). The isoclines indicate the combinations of parameters resulting in Δ = 5%, Δ = 10%, and Δ = 20%.

**Figure 5:**
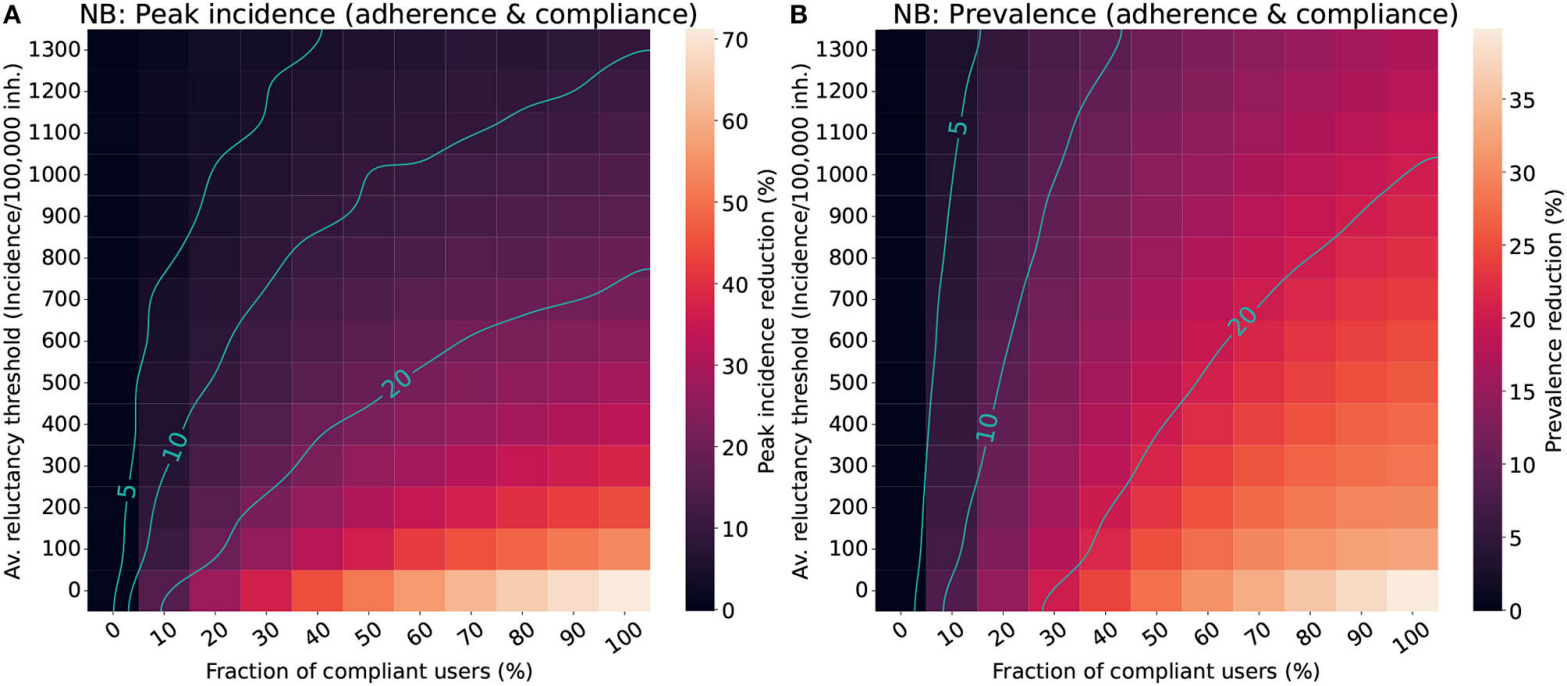
Impact of the population's average reluctance threshold and the level of compliance in the effectiveness of CT apps for a population with a Negative binomial distribution (NB). We assumed a maximal fraction of adopters of 70% for the “adherence & compliance” scenario. The color scale reflects the average reduction produced by the CT app (Δ) in the peak incidence **(A)** or maximal prevalence **(B)**. The isoclines indicate the combinations of parameters resulting in Δ = 5%, Δ = 10%, and Δ = 20%.

Figure 4 shows the results for the voluntary and imposed adoption scenarios and Figure 5 shows the results for the “adherence & compliance” simulation. The effectiveness of the apps is measured in terms of peak incidence reduction (Δ_*i*_) and prevalence reduction (Δ_*p*_). To facilitate the interpretation of the results we defined qualitative performance patterns depending on the incidence and prevalence reduction induced by the app: no effectiveness (Δ < 5%), low effectiveness (5% ≤ Δ < 10%), moderate effectiveness (10% ≤ Δ < 20%) and high effectiveness (Δ≥20%).

The analysis for the voluntary adoption scenario (Figures 4A, C) revealed that CT apps are only effective (Δ≥5%) when low reluctancy thresholds and high levels of adoption are present. Strategies with late adoption (*I*_thr_~1300 cases/100, 000 inh.) are only minimally effective in peak-incidence reduction when they are adopted by a very large fraction of users (>50%). However, late adoption is less relevant for prevalence reduction, where the same combination of parameters resulted in a moderate prevalence reduction (Δ_*p*_≥10%). Interestingly, to obtain a high peak incidence reduction (Δ_*i*_≥20%) it is not enough to have more than 50% of adopters, the population's average reluctancy threshold must also be very low (*I*_thr_ < 400 cases/100, 000 inh.).

In the imposed adoption scenario (Figures 4B, D) CT apps with high levels of adoption and moderate levels of compliance are necessary to obtain effective strategies (Δ≥5%). Apps with less than 10% of compliance are mostly ineffective, even when considering high levels of adoption. The relevance of compliance for app effectiveness is even more apparent when aiming toward a high-effectiveness strategy (Δ≥20%). Even when assuming a high level of adoption (50%), it is only possible to obtain a high peak incidence reduction if more than 30% of compliance is guaranteed.

The results for the “adherence & compliance” scenario (Figure 5) confirm the trends observed in the previous strategies, evidencing that moderate levels of compliance (>15%) and low reluctancy thresholds (*I*_thr_ < 800 cases/100, 000 inh.) are required to obtain relevant peak incidence reductions, and that even strategies with a high reluctancy threshold can result in moderate prevalence reductions if almost everyone complies with them.

The results of the sensitivity analysis to the population's degree distribution can be found in Supplementary Figures 4–7. Meanwhile, the analysis of different epidemic parameterization is shown in Supplementary Figures 8–12. The results obtained in all the supplementary scenarios evaluated support the conclusions extracted in the main results.

## 4. Discussion

The COVID-19 pandemic was one of the first cases in which digital contact tracing was widely used as an epidemic control measure. However, its empirical performance drastically differed from the one estimated in early modeling attempts. Health authorities in many countries have concluded that CT apps were completely ineffective, and in some cases, they even implied an extra burden for them.

One of the key lessons to be learned from COVID-19 pandemic is that the first models used to estimate the effectiveness of DCT systems were too simplistic. They modeled app adoption through a static point of view, assuming a constant amount of users for the whole duration of an outbreak. In reality, app adoption is a dynamic process, where individuals can decide to download and remove the app at will as the epidemic progresses. Heterogeneous reporting compliance has also been observed in empirical DCT systems, either because of the low willingness of an individual to report their status or the inability to do so due to technical issues.

These human behavior heterogeneities can impact the effectiveness of DCT systems but they have been disregarded in most prior DCT models. Our study proposes a novel approach for representing CT apps, where app adoption is modeled as a threshold dynamic depending on epidemic progression and heterogeneities in the reluctance threshold. Besides, we also include the effect of the level of compliance with infection reporting. Using this model we explored the interplay between app adoption and epidemic progression and characterized how three human behavioral heterogeneities alter the performance of CT apps. This was achieved by simulating three separate scenarios: the voluntary adoption (assuming complete compliance) the imposed adoption (assuming no reluctancy toward app adoption) and the “adherence & compliance” scenario, where the only constraint is the maximum number of people who can download the app (70% of the total population). An exploratory analysis of these scenarios allowed us to extract a set of recommendations (good practices) that may interest policy-makers when planning to use DCT systems in future outbreaks.

First, with the current maximal adoption levels (<30%) the effectiveness of CT apps is limited to only moderate or low effects. Thus, DCT systems should preferably be used as a complement to other mitigation strategies, such as classical contact tracing or social distancing measures. Prior research already identified the need for high levels of adoption to obtain effective strategies (33), and our analysis confirmed these outcomes. Apps adopted by less than 10-15% of the total population always result in ineffective strategies regardless of the time of adoption and the level of compliance assumed (Figure 4). This lower bound of adoption corresponds to approximately 21% of all smartphone users, a value very close to the minimal penetration needed identified in prior studies [20% in (5)].

Even with more than 50% of maximal adoption, high effectiveness strategies are only obtained when the reluctancy threshold is low (<400 cases/100,000 inh.) in the voluntary adoption scenario, or compliance is moderate or high (>20% compliant users). Note that incidences of the order of 1,000 cases/100,000 inh. were common in Europe through 2020-2022 and still app adoption was close to 20–30%. Similarly, even though people voluntarily downloaded the app, compliance was in the range of 20–40%, and thus one would expect lower values if adoption is imposed. As such, high effectiveness may not be reachable in empirical settings. Nonetheless, for large enough outbreaks—such as the one observed in the UK—even low reductions of prevalence may result in a noticeable decrease in hospital burden.

To aim toward highly effective strategies, policy-makers should emphasize the importance of early adoption and reporting compliance in their promotion campaigns. During the COVID-19 pandemic, the fast adoption of CT apps was mostly hindered by the public's privacy and data-sharing concerns (34). Policymakers should work to ensure that DCT systems are perceived as a safe and secure option way before the start of any new outbreak. This will probably imply a revision of the current implementations of DCT systems and massive promotion campaigns to establish them as safe and secure interventions.

Policy-makers should also focus on increasing reporting compliance. Limited research has assessed the importance of reporting compliance for DCT. An interesting study is Davis et al. (35), which explored how adherence and compliance can play a relevant role in a traditional contact-tracing strategy based on self-reporting. In their scenario with low self-reporting (around 11%), scalability did not have a significant impact on the overall effectiveness. This was also observed for our model, where increasing the percentage of adoption did not result in major improvements in app effectiveness for compliance levels below 20% (Figure 4D). Adherence only starts playing a major role in increasing app effectiveness if moderate levels of compliance are ensured.

Poor compliance may also be related to the very own implementation of the app. To exemplify this point, we analyzed the performance of RadarCOVID, the Spanish CT app (36). According to their statistics, after 40 weeks of implementation, the app had a 19% of penetration and only 6.7% of the users were able to report their infection (36). Our model shows that this combination of parameters results in a completely ineffective strategy both in terms of peak incidence and prevalence reduction (Figures 4B, D). The low levels of compliance in the Spanish app may have resulted from its complex reporting system. Users needed to obtain a verification code from the regional healthcare authorities and enter it into the CT app to report their infection. However, the codes were generated by the central Health Ministry, which had to communicate with regional authorities (37). Due to this interaction, there were significant delays in reporting infections, and many users did not receive their verification code even after requesting it.

We hypothesize that apps that follow a similar approach could benefit from a more straightforward reporting strategy, where the healthcare authorities are responsible for activating the positive status of an individual once they have provided their consent, removing the responsibility from the final user (38). This would ensure almost perfect compliance at the expense of maybe increasing privacy concerns toward app adoption. Hence, there is no perfect solution and more research should be devoted to understanding how DCT can actually be implemented in practice, taking into account the complexities of human behavior.

### 4.1. Limitations and future research

One of the main considerations of the study is the definition of the CT app model. App adoption was assumed to follow threshold dynamics only dependent on the prior incidence and the reluctance level of each individual. This follows the hypothesis that media reporting about disease progression can act as a driving force to encourage more reluctant individuals to download the app for their protection. There is still not a clear consensus about the main driving factor for app adoption. Guillon (39) suggests concerns about data protection and misinformation played a more relevant role in shaping app adoption than the perception of being at risk. However, Nguyen et al. (31), a study based on technology acceptance models (TAM), states that health risk perception has a positive effect on CT app adoption. Future research could aim toward developing an adoption model combining health risk perception and peer pressure dynamics. For generating such models it would be valuable to consult with policy-makers and implementation scientists as they can reveal other relevant factors that may condition the empirical performance of DCT systems.

Our app adoption model also assumes that infectees warn their contacts the same day of the diagnosis and the quarantine starts the exact day after the alert. Prior research has identified that delays in the reporting process, poor compliance with the preventive quarantine and testing unavailability can drastically diminish the effectiveness of CT app strategies (2, 40, 41). Empirical evidence also shows that even if compliance may be of the order of 40–70% of the users, only 10–50% of the ones that receive the alert contacted the authorities (8). In our case, we assumed that the latter was 100%.

Our epidemic model is not designed to precisely replicate the progression of the COVID-19 pandemic. Instead, its purpose is to assess the isolated impact of CT apps. Therefore, in our baseline simulations, we intentionally omit any consideration of the influence of other epidemic control interventions implemented during the COVID-19 pandemic (e.g., the use of face masks, social distancing, preventive screening, etc.), as well as any changes in the behavior of the population. This approach implies that our baseline scenario may yield higher incidence and prevalence values than those observed in real-world settings, as it does not account for the natural tendency of people to change their behavior during a pandemic, nor the effect of any of the non-pharmaceutical interventions that were implemented all over the world. Therefore, our results should be interpreted as an upper-bound estimate of the potential effectiveness of CT apps in realistic scenarios.

Future research could also be directed toward incorporating more realistic population structures into the model. It is known that age heterogeneities can play a major role in the distribution of reluctance toward app adoption (5) and also in the average number of in-person contacts. Thus, it would be possible to modify the distribution of reluctance to better describe the patterns observed in realistic populations. Prior studies have reported that a high CT app coverage amongst adults plays a central role in preventing transmission to older adults, who have less accessibility to smartphones (5). By including age heterogeneities in our model it will also be possible to use more complex epidemic models that consider the distinction between individuals who die and recover from the disease. This will provide some insight into the number of deaths averted by the use of CT apps among the different age groups.

### 4.2. Conclusion

Overall, this study presents a novel approach to represent the co-evolution of epidemic progression and CT app adoption from a dynamic point of view. This approach allowed us to better characterize CT apps by including human behavioral heterogeneities like the individual's reluctance toward app adoption or different levels of compliance.

With this approach, we identified some relevant “good practices” that should be followed by policy-makers aiming to use DCT systems in new epidemic outbreaks. The summarized recommendations are the following:

- The first models of DCT systems were far too optimistic because they disregarded several crucial factors of human behavior.
- Even if high levels of adoption are needed to obtain highly effective CT apps, there are other important factors to consider, like an early adoption and at least moderate levels of compliance.
- CT apps can hardly be used as the only protective measure during an outbreak. Instead, they are better used to complement other mitigation strategies.
- The fast adoption of DCT systems should be prioritized in future outbreaks.
- Reporting compliance has been one of the main bottlenecks of some of the CT apps implemented during the COVID-19 pandemic.
- Low compliance can derive from an overly complicated design of the app's reporting system.
- A reporting system that removes the responsibility for reporting from the final user may aid in increasing compliance.
- More research should be directed toward integrating the complexities of human behavior in epidemic processes, especially when planning interventions for epidemic control. These models should incorporate heterogeneity in adherence and compliance with the interventions.

One way in which policy-makers could apply these recommendations is by promoting the development of DCT systems in non-pandemic periods. This proactive approach would give time to the general public to trust such systems before they must be used, likely reducing the population's average reluctance once the outbreak starts. Another advantage of this method is that CT apps could be easily activated under demand. This would lead to shorter delays between disease onset and CT app implementation, which is one of the key factors needed to boost their effectiveness.

Even with these considerations, it is likely that DCT systems cannot be the sole solution for epidemic control. We observed that their limited performance in realistic settings makes them more suited to become a complement to other traditional epidemic control strategies.

## Data availability statement

Publicly available datasets were analyzed in this study. This data can be found at: https://doi.org/10.1038/s41467-020-20544-y and https://doi.org/10.1371/journal.pmed.0050074.

## Author contributions

AF: Formal analysis, Investigation, Software, Visualization, Writing—original draft, Writing—review & editing. AA: Conceptualization, Writing—review & editing. YM: Conceptualization, Project administration, Supervision, Writing—review & editing.
