# Supplementary material for: Characterizing the role of human behavior in the effectiveness of contact-tracing applications
Source: Front Public Health. 2023 Nov 3;11:1266989. doi: 10.3389/fpubh.2023.1266989 (PMC10657191; doi:10.3389/fpubh.2023.1266989)
Supplement: Supplementary file 1 [file Data_Sheet_1.pdf]

Supplementary Material

1 SUPPLEMENTARY METHODS

1.1 Networks

Ref.[1] identified that the degree distribution of risk contacts in an epidemic contagion scenario of an air-borne disease follows a negative binomial distribution. Thus, the degree distribution of our realistic population was assumed to follow this distribution with the shape parameters (n and p) parameterised according to survey data. A negative binomial distribution is usually described through the probability mass function

$$f(k) = \binom{k+r-1}{k} p^n (1-p)^k \tag{S1}$$

where r is the number of successes ($r \geq 0$), k is the number of failures, and p is the probability of a single success. The distribution can also be parameterised in terms of the mean number of failures (μ) needed to succeed [2], by defining the probability of success (p) as

$$p = \frac{r}{r+\mu}. \tag{S2}$$

Through this definition, the distribution can be completely characterised by only fitting r and μ. In our case, μ is the average number of contacts of each individual, their average degree ($\langle k \rangle$). We estimated it from the age-mixing matrices for the Italian population described in Ref.[3]. As we did not consider age heterogeneities in our analysis, the population's average degree was defined as the average number of contacts across all age groups ($\mu = 11.85$). For estimating the r parameter, we fit a negative binomial distribution with $\mu = 11.85$ using the survey data from the POLYMOD study [1]. The fitting process was performed in R (version 4.2.1), using the approach proposed in Ref [4] and it allowed us to identify that $r = 2.426$ (see figure S1).

To define the degree of each node we used the function *nbinom.rvs()* from the Python package *SciPy* (version 1.7.3) [5] to draw 10,000 random samples from the estimated degree distribution. Nonetheless, in epidemic processes, the disease can only spread if $k > 2$. To model this effect while maintaining the desired $\langle k \rangle$ we performed the sampling process using a negative binomial distribution with

$$\begin{aligned} r &= 2.426, \\ \mu^* &= \mu - k_{min}, \\ p &= \frac{n}{n+\mu^* - k_{min}}. \end{aligned} \tag{S3}$$

where $k_{min} = 2$. The distribution obtained has the same shape as the negative binomial extracted from the survey data but with $\mu^* = \mu - 2$. In this way, we can sum $k_{min} = 2$ to all the sampled values, to obtain the desired $\langle k \rangle$ while meeting the k_{min} criteria. Finally, the list of degrees was transformed into a graph using the configurational model [6] with the Fabien Viger implementation [7], which creates undirected, connected, simple graphs while respecting the desired degree distribution.

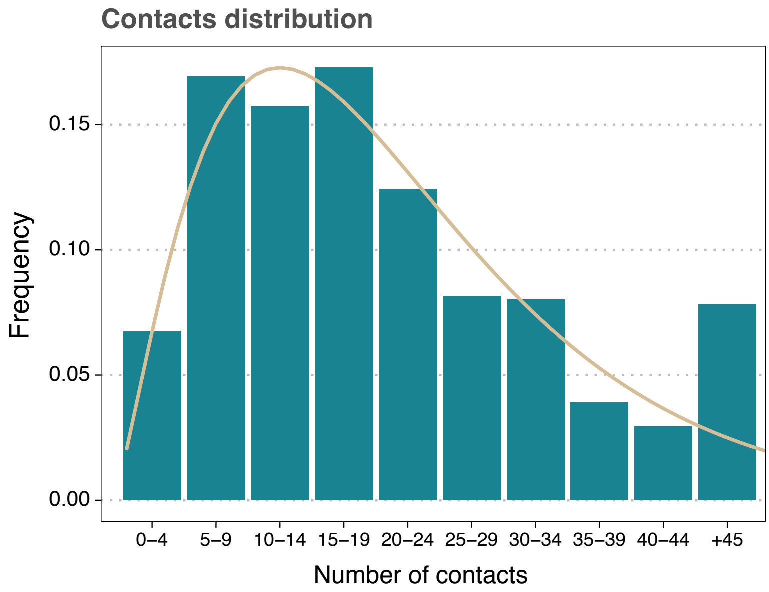

Figure S1. Fit of the survey data from Ref.[1]. The best fit is obtained using a Negative Binomial distribution with $\mu = 11.85$ and $r = 2.426$.

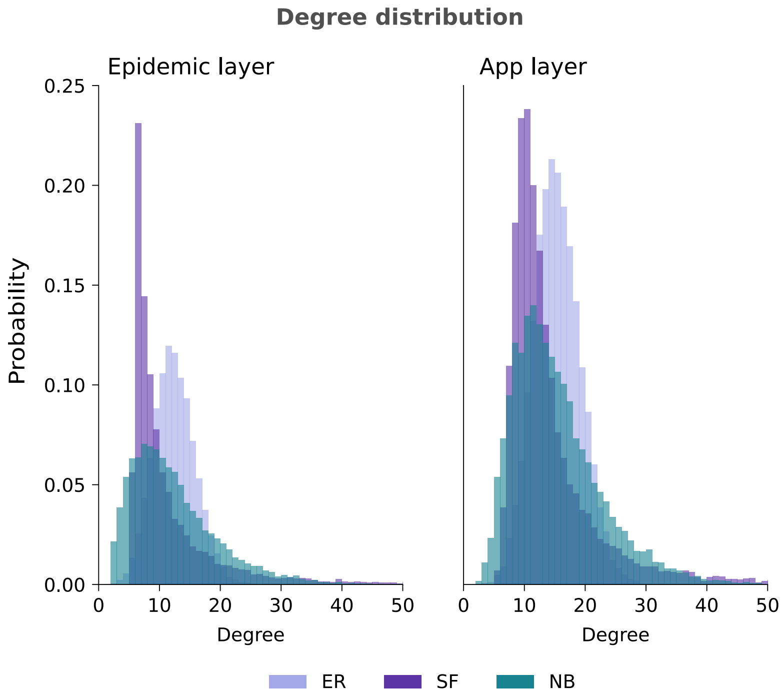

Figure S2. Comparison of the degree distributions of the networks in the epidemic (left-panel) and CT app (right-panel) layers. The three distributions reflect respectively, an Erdős-Rényi random graph (ER), a Scale-free network (SF) and a Negative Binomial degree distribution fit with survey data (NB).

To test the generality of the results we repeated the analysis using two artificial degree distributions with the same $\langle k \rangle$, an Erdős-Rényi [8] random graph and a Scale-Free network [6]. Erdős-Rényi [8] random

graphs reflect homogeneous mixing in a population. All individuals have the same probability ($p = \frac{1}{N}$) to interact with others. The Erdős-Rényi network has a degree distribution with a binomial shape centred on the $\langle k \rangle$. The distribution of the Scale-Free network follows a power law with an exponent $\alpha_{PL} = 2.5$ and the x_{min} parameter was tuned to produce a distribution with the desired average degree. To generate a scale-free network with uncorrelated degree nodes, the maximal degree of the network was constrained to

$$k_{max} = \sqrt{N} \tag{S4}$$

where N is the number of individuals in the network [9]. To this end, nodes with a degree $> k_{max}$ were re-sampled until the constraint was met. As for the negative binomial distribution, the connectivity pattern was also generated using the configurational model implemented in the *igraph* package (version 0.9.8) [7].

The connectivity pattern in the CT app network was generated by expanding the network on top of which the epidemic spreads with 25% of random interactions, which represent spurious interactions misinterpreted by the CT app as risk contacts. To create them, we generated a large pool of potential interactions that are not already present in the network and are not self-loops. We sampled the desired number of edges from the pool and then included them in the original network. This process was performed in the same way for the three population networks.

The degree distributions obtained in the three population structures are shown in figure S2.

1.2 Epidemic modelling terms

We have incorporated a more detailed description of some of the epidemic modelling terms used in this manuscript (Table S1). This should facilitate the interpretation of the Methods applied in this study.

Term	Symbol	Definition	Source
Basic reproductive number	R_0	Number of secondary infections caused by a single infective introduced into a wholly susceptible population.	[10]
Incubation period	IP	Period between exposure to an infectious agent and the onset of symptoms of the disease.	[6, 10, 11]
Susceptible	S	Individuals who can become infected if they contact a contagious individual (healthy individuals).	[6, 10, 11]
Exposed	E	Individual who has become infected but are not yet infectious. The disease is in its latent state.	[11]
Pre-symptomatic infectious	P	Disease stage in which individuals exhibit no symptoms, but they can transmit the disease.	[10, 11]
Symptomatic infectious	I	Individuals who are infectious and show symptoms of the disease.	[11]
Removed	R	Individuals who have lost contagious capacity, either due to natural causes (recovery or death) or due to behavioural changes (reduced social contacts).	[6, 10, 11]
Generation time	Tg	Interval between infector–infectee pairs. The average Tg can be defined as the sum of the average latent (ϵ) and the average infectious period ($\rho + \mu$).	[10, 12, 13]
Incidence	-	Number of new infections over a period of time.	[10, 13]
Prevalence	-	Cumulative number of cases of a disease.	[10, 13]

Table S1. Definitions of the epidemic modelling terms used across the whole manuscript.

1.3 Parameters

Table S2 shows the complete list of all the parameters of the epidemic model, the CT app adoption dynamics, the networks used for the analysis and the simulations. Parameters marked with a ”-” are the three human behavioural parameters characterised by our analysis.

PARAMETERS OF THE DYNAMICAL MODEL

Epidemic model		CT app model	
I_0	0.0005	App_0	0.01
β	0.045	I_{thr}	-
ϵ	1/3	Percentage of compliant users	-
ρ	1/2	α	1/10
μ	1/2	Max percentage of adoption	-
Leave quarantine prob.	1/10		
Detection rate	0.5		

PARAMETERS OF THE NETWORK STRUCTURE

Epidemic network		CT app network	
Number of nodes	10,000	Number of nodes	10,000
$\langle k_{ER} \rangle$	11.93	$\langle k_{ER} \rangle$	14.92
$\langle k_{SF} \rangle$	11.92	$\langle k_{SF} \rangle$	14.90
α_{PL}	2.5	α_{PL}	2.5
x_{min} SF	5.3	x_{min} SF	10.45
$\langle k_{NB} \rangle$	11.85	$\langle k_{NB} \rangle$	14.82
r_{NB}	2.426		

PARAMETERS OF THE SIMULATIONS

time-steps (days)	500
Repetitions	1000

Table S2. Summary of all the parameters used in the epidemic-CT app model. The average reluctancy threshold, percentage of compliant users and maximal percentage of adoption are the quantities explored in the analysis.

1.4 Alignment of repetitions

The 1000 repetitions of each simulation were aligned before estimating the average response and the confidence interval (CI). This ensures that the average is always estimated over an equivalent point in disease progression, regardless of the delay in the emergence of the outbreak induced by the stochastic nature of the simulations. The alignment process followed the approach described in Ref. [14], where $t = 0$ is defined at the time-step where all outbreaks have 1% of the total population infected. This process modifies the temporal overlap of the simulations, generating some non-overlapping regions at both ends. To address it, we padded all simulations with their edge values until the full temporal range was complete.

1.5 7-day incidence estimation

We reported the temporal evolution of the epidemic and app adoption dynamics using the average results for the last 7 days. This process was performed by using the convolution of the incidence, prevalence and app adoption trends with a uniform window of 7 time steps (7 days). The convolution process allows us to reduce the noise on the incidence trends and to provide a more realistic representation of incidence progression, which usually is not reported daily.

2 SUPPLEMENTARY RESULTS

2.1 Temporal evolution for different parameters

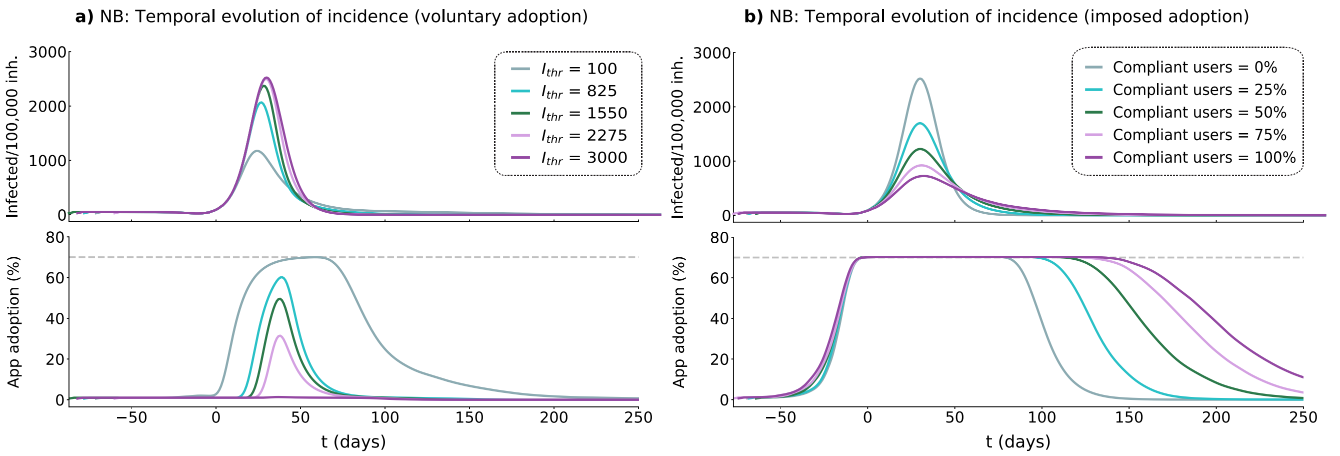

Figure S3. Temporal evolution of the epidemic and CT app adoption dynamics for increasing values of the driving parameter of each scenario. **(A)** Changes in the incidence and the percentage of app adoption for reluctancy thresholds in the $100 < I_{\text{thr}} < 3000$ range. **(B)** Changes in the incidence and the percentage of app adoption for percentages of compliance ranging from 0 to 100%.

Figure S3 shows the differences in the progression of the epidemic and CT app adoption dynamics for different reluctancy thresholds (figure S3A) and percentage of compliant users (figure S3B). We observe that CT apps introduced early in disease progression (low threshold) result in a higher peak of adoption and thus they induce a flattening of the epidemic curve. Additionally, CT apps with a lower threshold are adopted for a wider time window, also contributing to their effectiveness. Contrarily, CT apps introduced very close to the epidemic peak (or after it) almost have no effect in flattening the epidemic curve.

Changes in compliance do not affect directly the app adoption process, they only modify the effectiveness of the reporting system. However, the coupling between disease progression and app adoption induces some indirect effects. The app adoption dynamic grows in the same way for all compliance levels, reaching almost simultaneously the maximal level of adopters (70%). However, we do observe that modifying compliance alters the time of removal of the CT app. This indirect effect is derived from a change in the flattening of the epidemic curve produced by the CT app. In this scenario, if the epidemic curve is flatter and lasts for longer, users maintain the app downloaded for a longer period, which also affects the performance of the strategy.

2.2 Exploratory analysis of the human behavioural parameters for different network structures

Figure S4 and figure S5 show the exploratory analysis for the voluntary and imposed adoption scenarios in a population with an Erdős-Rényi and a Scale-Free degree distributions, respectively. The equivalent results for the "adherence & compliance" scenario can be found in figure S6 and figure S7. The results obtained are consistent with the ones for the realistic population (negative binomial distribution). High adoption and moderate levels of compliance are crucial for effective strategies. Additionally, we observe that the random population has a very similar profile to the negative binomial network, with a slightly

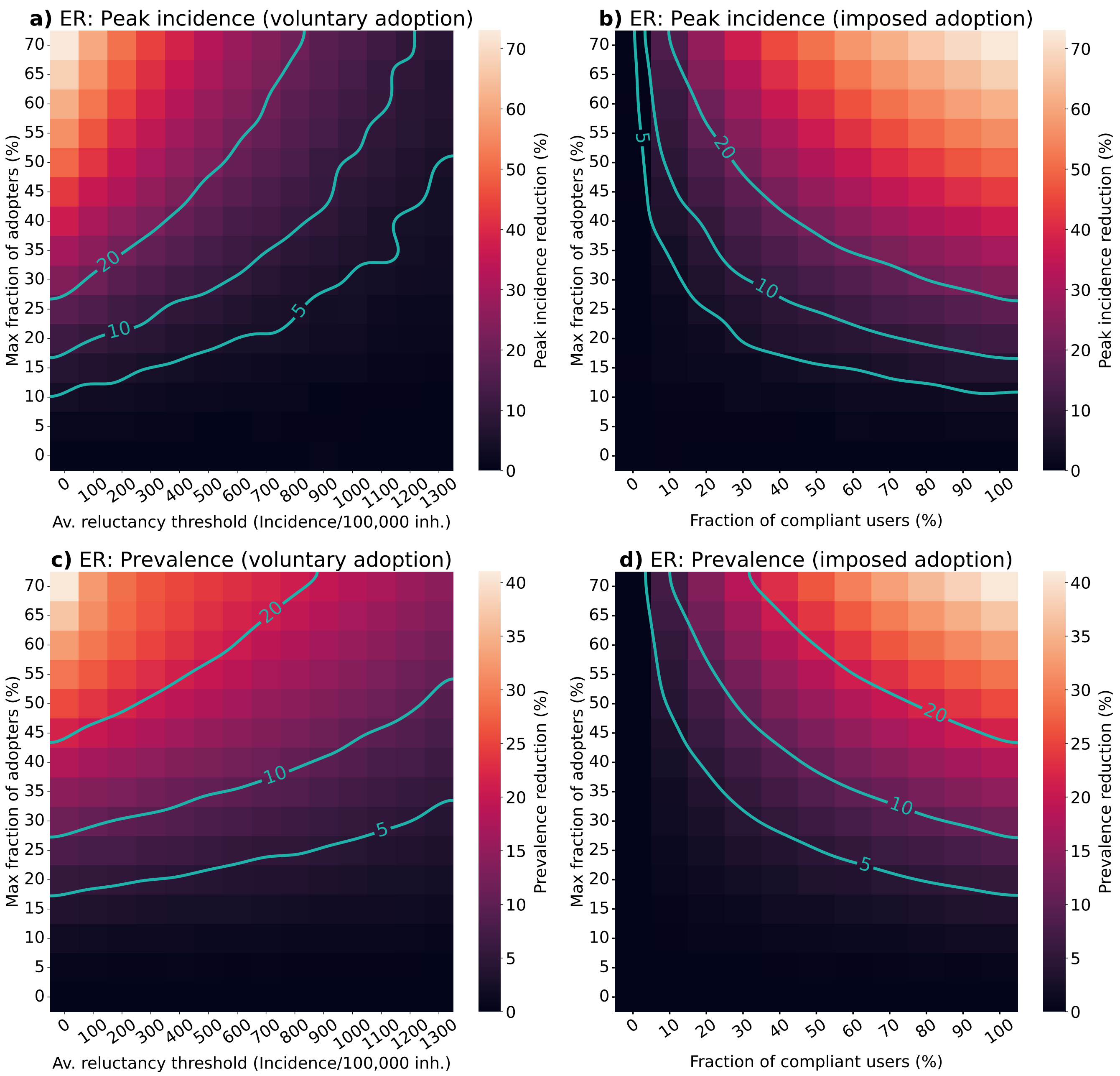

Figure S4. Impact of different factors of human behaviour in the effectiveness of CT apps in a population with an Erdős-Rényi random graph structure. For the voluntary adoption scenario (panels **(A)** and **(C)**), the parameters explored are the average reluctancy threshold and the maximal fraction of adopters, while in the imposed adoption scenario (panels **(B)** and **(D)**), changes in the fraction of compliant users and the maximal fraction of adopters are explored. The colour scale reflects the average reduction produced by the CT app (Δ) in the peak incidence (top panels) or maximal prevalence (lower panels). The isoclines indicate the regions with $\Delta = 5\%$, $\Delta = 10\%$ and $\Delta = 20\%$.

wider effective parameter space than in the realistic case. Contrarily, the Scale-free population shows a very narrow parameter space with effective apps. This may result from the increased transmissibility in the heterogeneous population, which diminishes the effectiveness of the strategy.

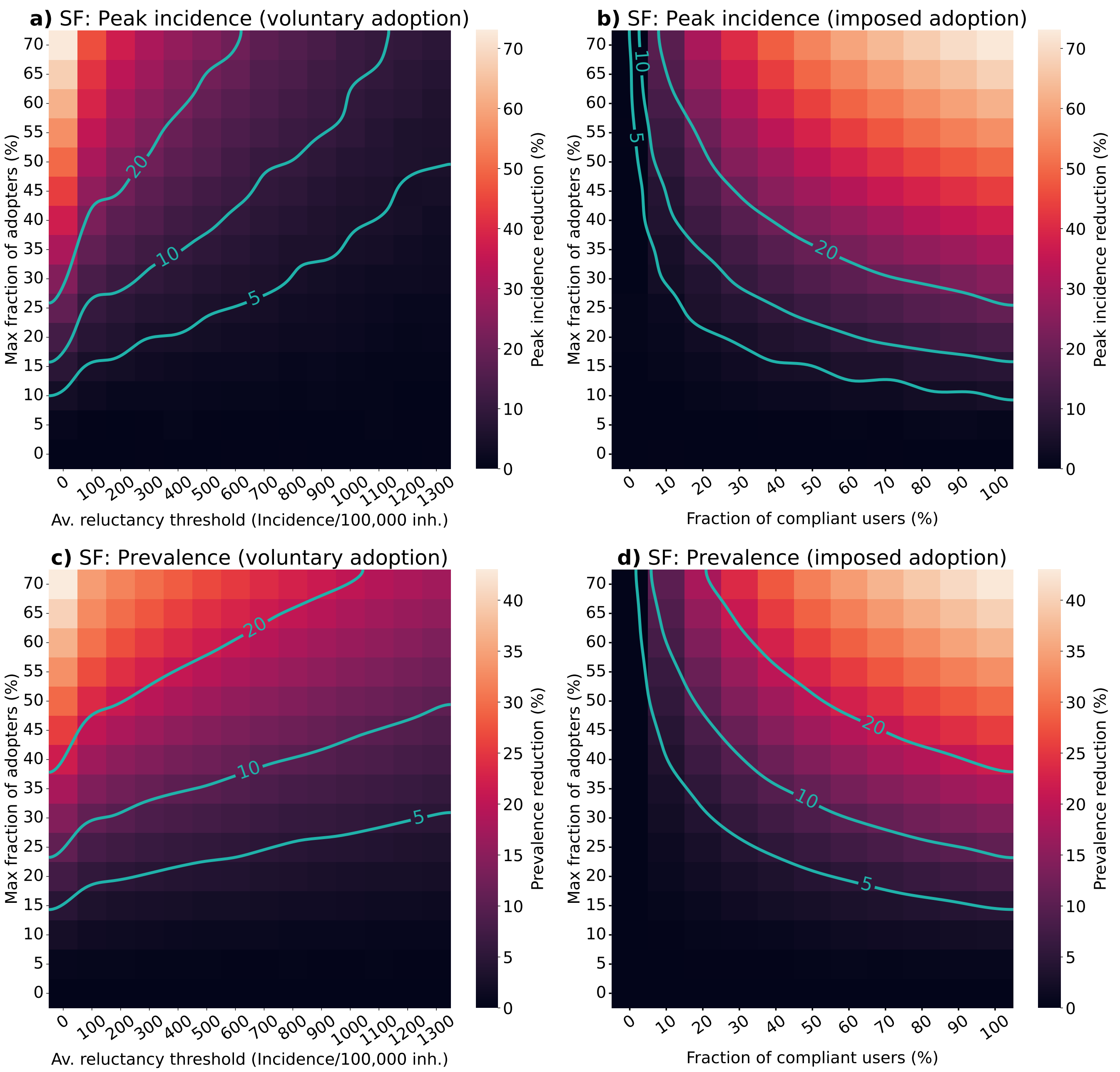

Figure S5. Impact of different factors of human behaviour in the effectiveness of CT apps in a population with a Scale-Free degree distribution. For the voluntary adoption scenario (panels **(A)** and **(C)**), the parameters explored are the average reluctancy threshold and the maximal fraction of adopters, while in the imposed adoption scenario (panels **(B)** and **(D)**), changes in the fraction of compliant users and the maximal fraction of adopters are explored. The colour scale reflects the average reduction produced by the CT app (Δ) in the peak incidence (top panels) or maximal prevalence (lower panels). The isoclines indicate the regions with $\Delta = 5\%$, $\Delta = 10\%$ and $\Delta = 20\%$.

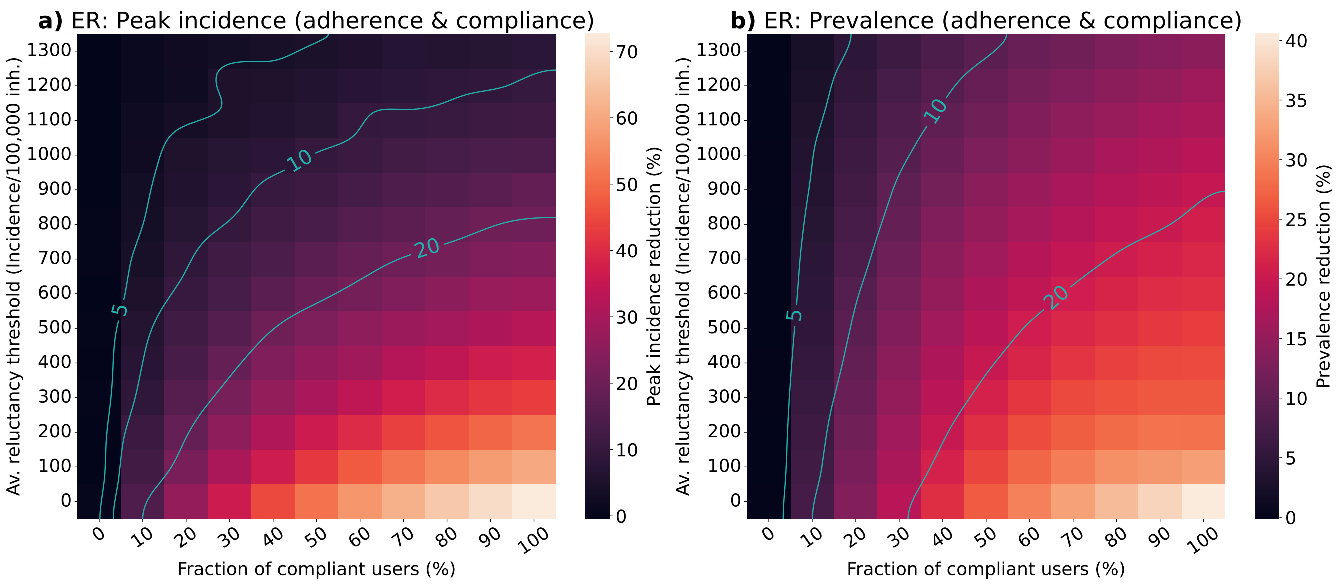

Figure S6. Impact of the population's average reluctancy threshold and the reporting compliance in the effectiveness of CT apps for a population with an Erdős-Rényi distribution. This scenario ("adherence & compliance") follows the assumption that $max(App) = 70\%$. **(A)** shows the effectiveness of the CT app (Δ) in terms of average peak incidence reduction, while for **(B)** the effectiveness in terms of prevalence is observed. The isoclines indicate the regions with $\Delta = 5\%$, $\Delta = 10\%$ and $\Delta = 20\%$.

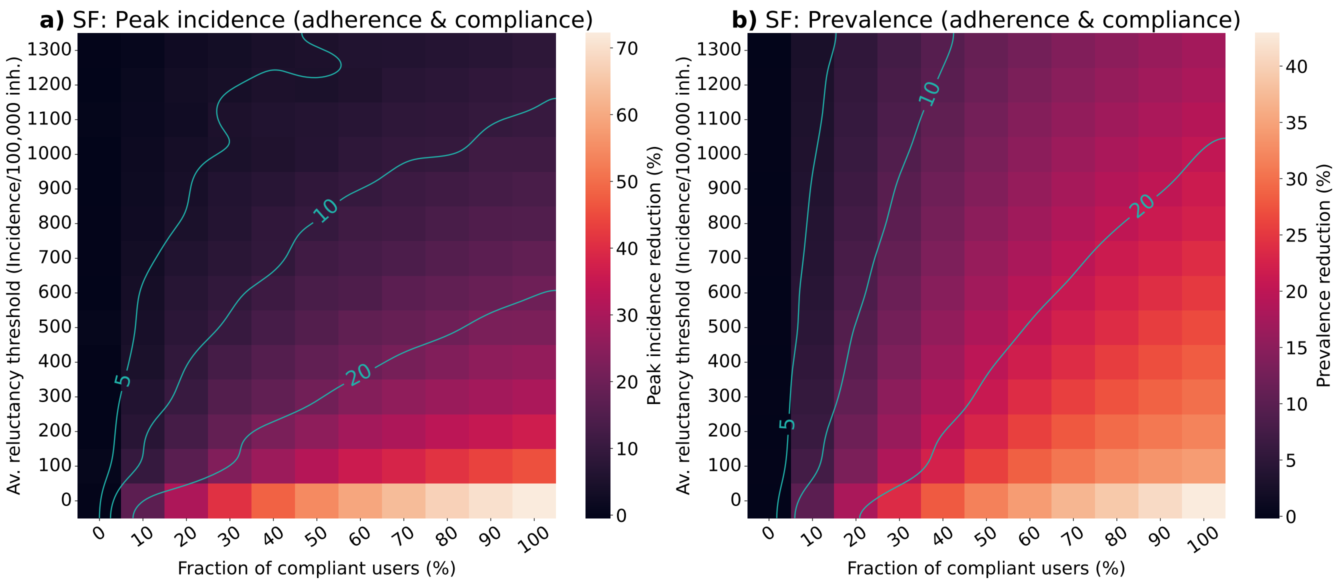

Figure S7. Impact of the population's average reluctancy threshold and the level of compliance in the effectiveness of CT apps for a population with a Scale-Free distribution. This scenario ("adherence & compliance") follows the assumption that $max(App) = 70\%$. **(A)** shows the effectiveness of the CT app (Δ) in terms of average peak incidence reduction, while for **(B)** the effectiveness in terms of prevalence is observed. The isoclines indicate the regions with $\Delta = 5\%$, $\Delta = 10\%$ and $\Delta = 20\%$.

2.3 Exploration of different epidemic scenarios

To validate the generality of our recommendations, we assessed the performance of the CT app with two different epidemic parametrisations. Both parametrisations have the same β as in the original parametrisation ($\beta = 0.045$) but they assume a shorter incubation period (3.5 days), similar to what was reported during the Omicron phase [15]. Each scenario then assumes a different removal probability (μ), resulting in remarkably different R_0 values (see Fig. S8).

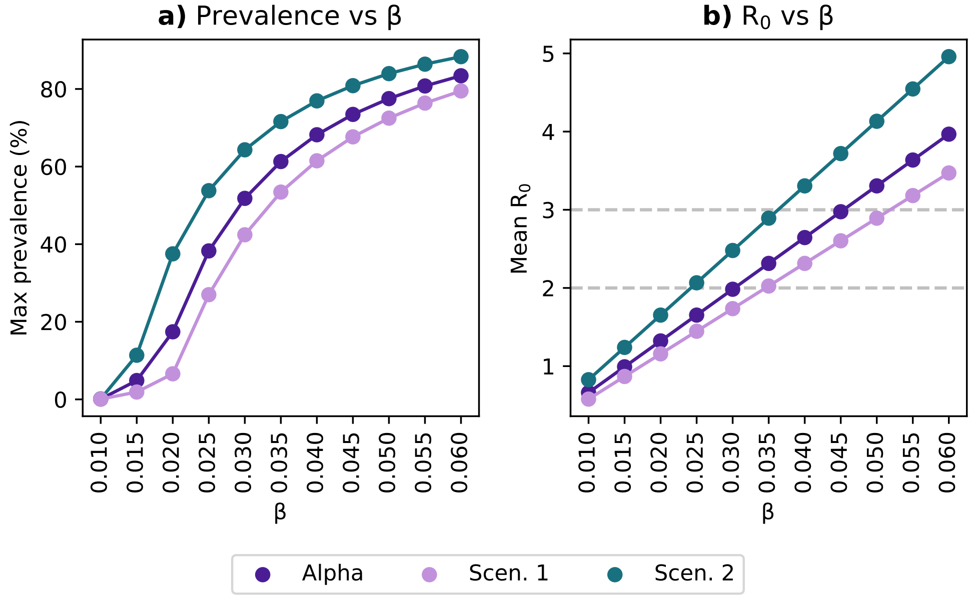

Figure S8. R_0 estimations for the three epidemic scenarios evaluated. **(A)** Maximal prevalence obtained for each value of β. **(B)** Basic reproductive number estimated for each value of β. Purple trends represent the results for the original formulation of the model, reflecting the Alpha variant of Covid-19 (estimated $R_0 = 3$). Light violet trends (Scenario 1) reflect an epidemic outbreak with an incubation period of 3.5 days and a removal probability $\mu = 1/2$. For $\beta = 0.045$ it results in $R_0 = 2.6$. Blue trends (Scenario 2) reflect an epidemic outbreak with 3.5 days of incubation period and a removal probability $\mu = 1/3.5$. For $\beta = 0.045$ it results in $R_0 = 3.7$. All models were tested on the population structure with a negative binomial distribution obtained from survey data.

The scenarios evaluated have the following specifications:

1. **Scenario 1:** Incubation period of 3.5 days and same removal probability as in the alpha variant ($\mu = 1/2$). This scenario results in an R_0 of 2.6. Parameters: $\beta = 0.045$, $\epsilon = 1/2$, $\rho = 1/1.5$ and $\mu = 1/2$.
2. **Scenario 2:** Incubation period of 3.5 days and lower removal probability than in the alpha variant ($\mu = 1/3.5$). This scenario results in an R_0 of 3.7. Parameters: $\beta = 0.045$, $\epsilon = 1/2$, $\rho = 1/1.5$ and $\mu = 1/3.5$.

The results obtained show that human behaviour similarly affects CT apps regardless of the epidemic parameters assumed (see Fig. S9-S12). Apps adopted by less than 10-15% of the total population are also ineffective in both scenarios, regardless of the time of adoption and the level of compliance assumed. Moreover, even with high levels of maximal penetration ($> 50\%$) low reluctance and moderate levels of compliance are necessary to obtain effective interventions ($> 20\%$ compliance).

Even if the general conclusions of our original formulation are preserved, we do observe a slight increase in the maximal effectiveness of the CT app. The original formulation (alpha variant of Covid-19) achieved a maximal peak incidence reduction of 72% and a prevalence reduction of 40%. Meanwhile, with a lower incubation period, scenarios 1 and 2 reached, respectively, a maximal peak incidence reduction of 77% and 74%, and a prevalence reduction of 41% and 47%.

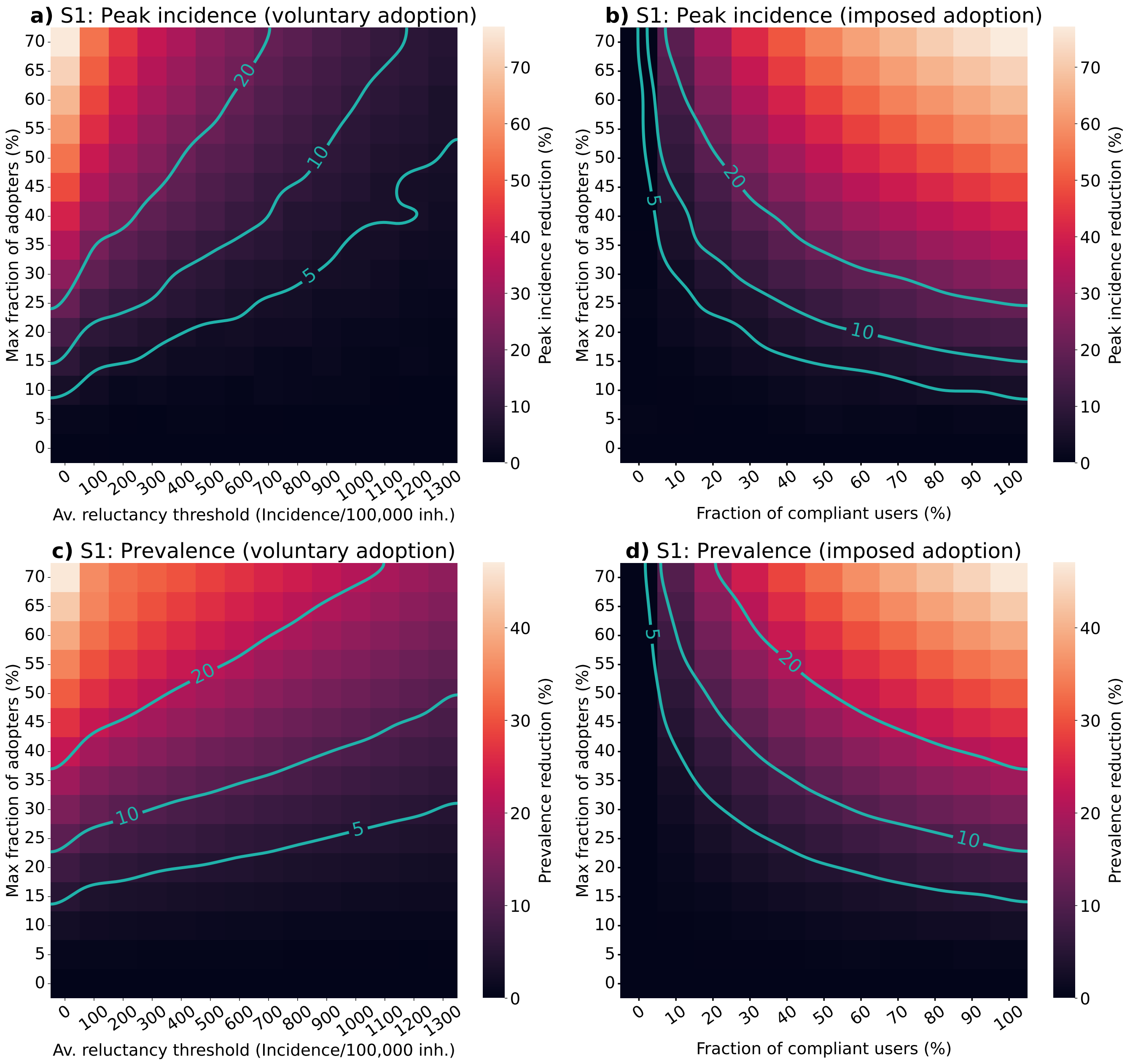

Figure S9. Epidemic scenario 1: Incubation period of 3.5 days and a removal probability of $\mu = 1/2$ ($R_0 = 2.6$). The population follows a Negative binomial distribution fit with survey data. For the voluntary adoption scenario (panels **(A)** and **(C)**), the parameters explored are the average reluctancy threshold and the maximal fraction of adopters, while in the imposed adoption scenario (panels **(B)** and **(D)**), changes in the fraction of compliant users and the maximal fraction of adopters are explored. The colour scale reflects the average reduction produced by the CT app (Δ) in the peak incidence (top panels) or maximal prevalence (lower panels). The isoclines indicate the regions with $\Delta = 5\%$, $\Delta = 10\%$ and $\Delta = 20\%$.

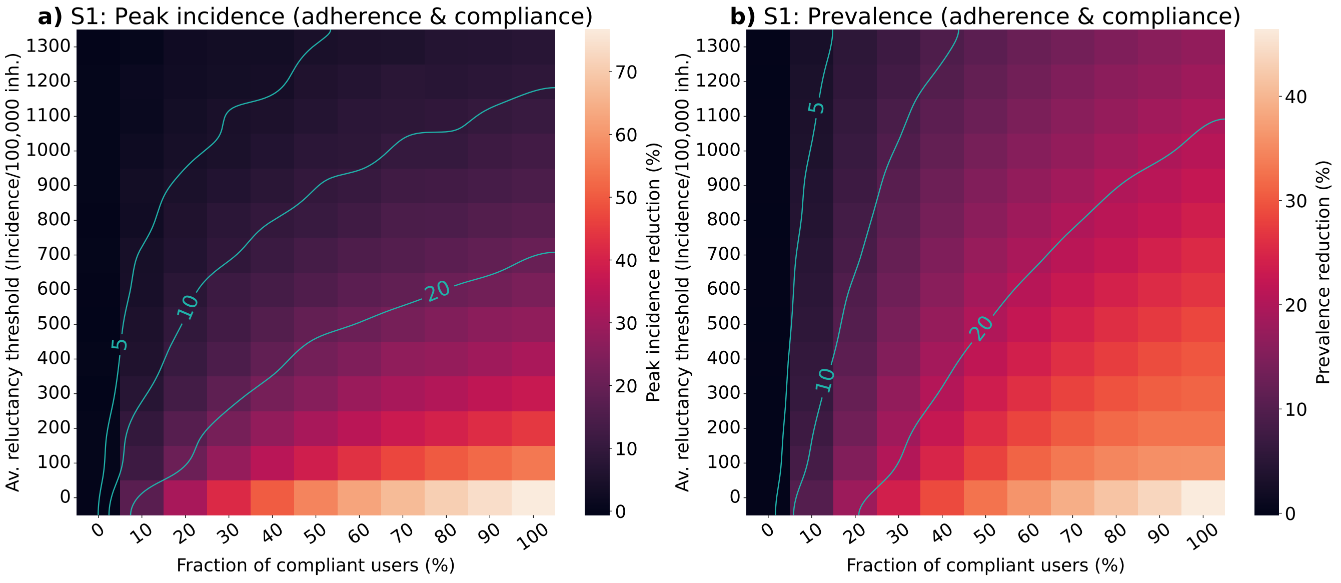

Figure S10. Epidemic scenario 1: Incubation period of 3.5 days and a removal probability of $\mu = 1/2$ ($R_0 = 2.6$).The population follows a Negative binomial distribution fit with survey data. The "adherence & compliance" scenario follows the assumption that $max(App) = 70\%$. **(A)** shows the effectiveness of the CT app (Δ) in terms of average peak incidence reduction, while for **(B)** the effectiveness in terms of prevalence is observed. The isoclines indicate the regions with $\Delta = 5\%$, $\Delta = 10\%$ and $\Delta = 20\%$.

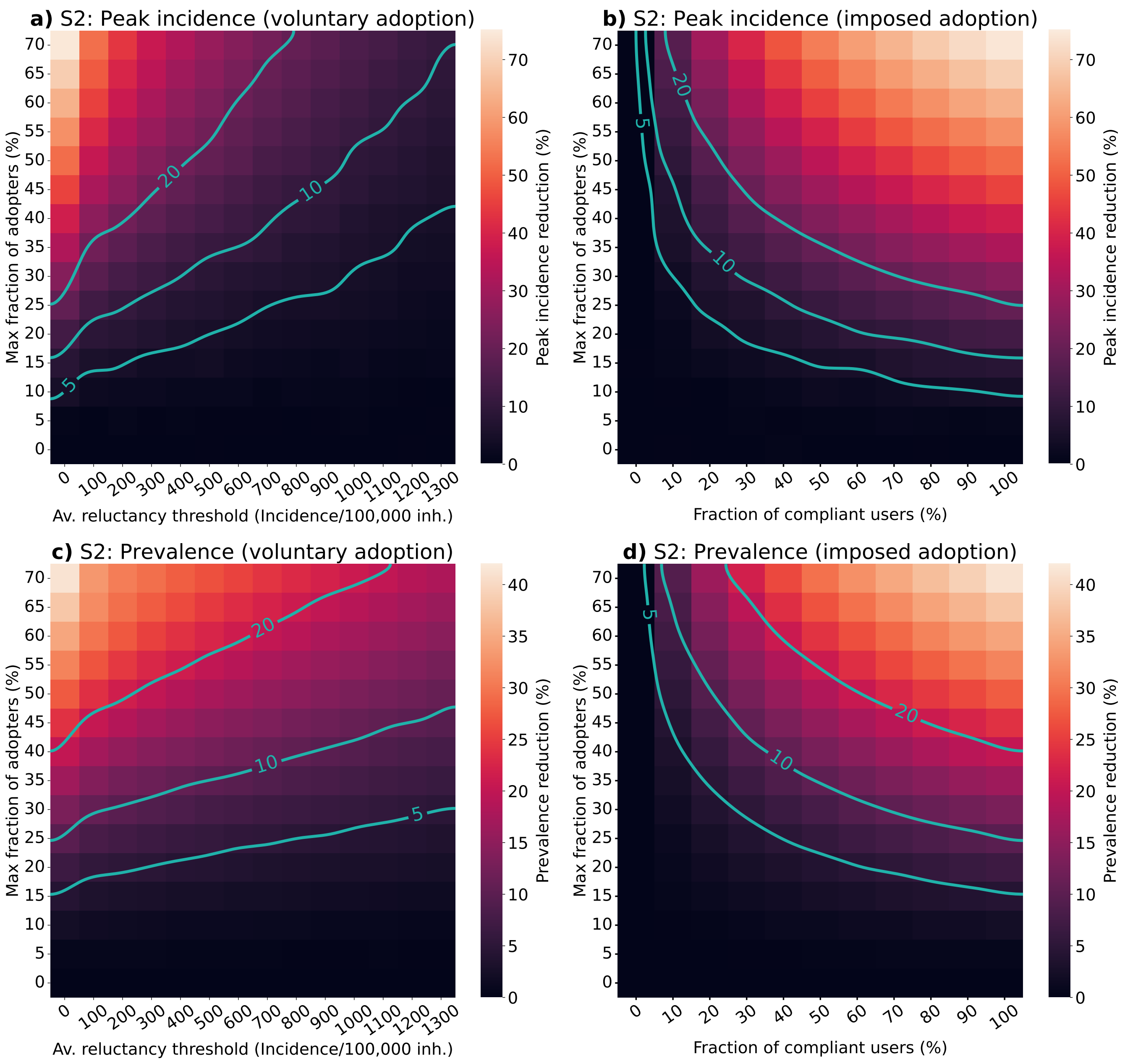

Figure S11. Epidemic scenario 2: Incubation period of 3.5 days and a removal probability of $\mu = 1/3.5$ ($R_0 = 3.7$). The population follows a Negative binomial distribution fit with survey data. For the voluntary adoption scenario (panels **(A)** and **(C)**), the parameters explored are the average reluctancy threshold and the maximal fraction of adopters, while in the imposed adoption scenario (panels **(B)** and **(D)**), changes in the fraction of compliant users and the maximal fraction of adopters are explored. The colour scale reflects the average reduction produced by the CT app (Δ) in the peak incidence (top panels) or maximal prevalence (lower panels). The isoclines indicate the regions with $\Delta = 5\%$, $\Delta = 10\%$ and $\Delta = 20\%$.

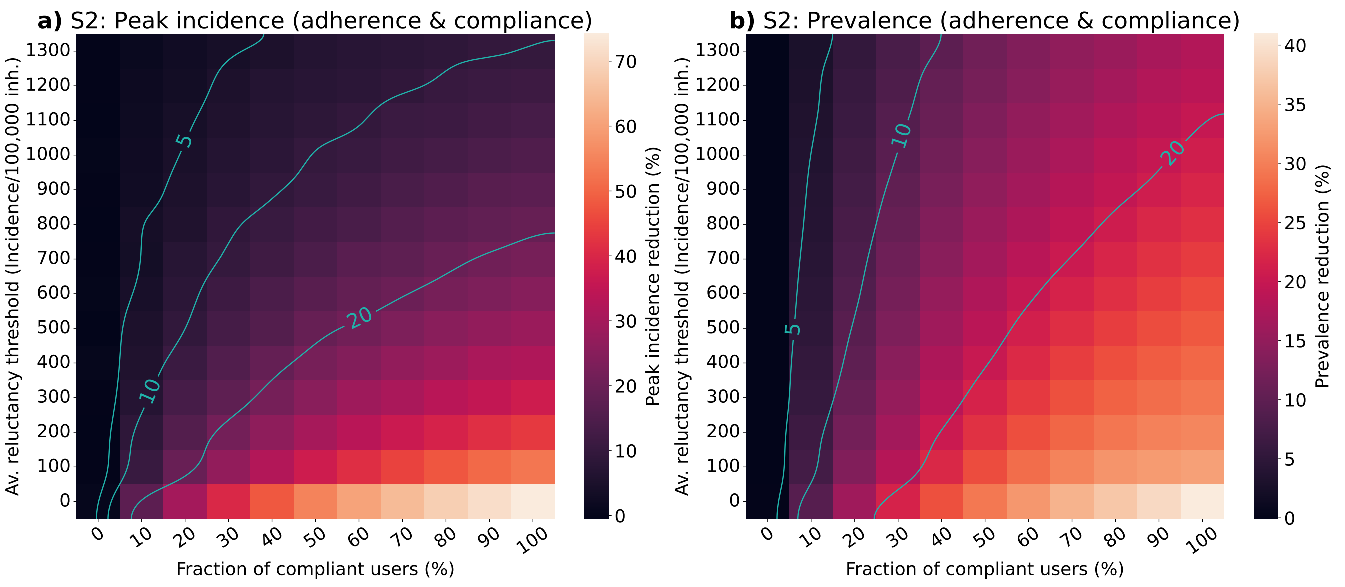

Figure S12. Epidemic scenario 2: Incubation period of 3.5 days and a removal probability of $\mu = 1/3.5$ ($R_0 = 3.7$). The population follows a Negative binomial distribution fit with survey data. The "adherence & compliance" scenario follows the assumption that $max(App) = 70\%$. **(A)** shows the effectiveness of the CT app (Δ) in terms of average peak incidence reduction, while for **(B)** the effectiveness in terms of prevalence is observed. The isoclines indicate the regions with $\Delta = 5\%$, $\Delta = 10\%$ and $\Delta = 20\%$.

REFERENCES

[1]Mossong J, Hens N, Jit M, Beutels P, Auranen K, Mikolajczyk R, et al. Social contacts and mixing patterns relevant to the spread of infectious diseases. *PLoS Med* **5** (2008) e74. doi:10.1371/journal.pmed.0050074.

[2]Cook JD. Notes on the negative binomial distribution. *Unknow, October* **28** (2009) 2009.

[3]Mistry D, Litvinova M, y Piontti AP, Chinazzi M, Fumanelli L, Gomes MF, et al. Inferring high-resolution human mixing patterns for disease modeling. *Nature communications* **12** (2021) 1–12. doi:10.1038/s41467-020-20544-y.

[4]Lu D, Aleta A, Ajelli M, Pastor-Satorras R, Vespignani A, Moreno Y. Data-driven estimate of SARS-CoV-2 herd immunity threshold in populations with individual contact pattern variations. *medRxiv* (2021). doi:10.1101/2021.03.19.21253974.

[5]Virtanen P, Gommers R, Oliphant TE, Haberland M, Reddy T, Cournapeau D, et al. SciPy 1.0: Fundamental Algorithms for Scientific Computing in Python. *Nature Methods* **17** (2020) 261–272. doi:10.1038/s41592-019-0686-2.

[6]Barabási AL. *Network science* (Cambridge: Cambridge University Press) (2015).

[7]Csardi G, Nepusz T. The igraph software package for complex network research. *InterJournal* (2006) 1695.

[8]Erdős P, Rényi A. On the evolution of random graphs. *Publ. Math. Inst. Hung. Acad. Sci* **5** (1960) 17–60. doi:10.1515/9781400841356.38.

[9]Catanzaro M, Boguná M, Pastor-Satorras R. Generation of uncorrelated random scale-free networks. *Physical review e* **71** (2005) 027103. doi:10.1103/PhysRevE.71.027103.

[10]Moghadas S, Milwid R. Glossary of terms for infectious disease modelling: a proposal for consistent language. *Winnipeg, MB: NCCID* (2016) 1–3.

[11]Martcheva M, Martcheva M. Introduction to epidemic modeling. *An introduction to mathematical epidemiology* (2015) 9–31.

[12]Svensson Å. A note on generation times in epidemic models. *Mathematical biosciences* **208** (2007) 300–311. doi:10.1016/j.mbs.2006.10.010.

[13]Anderson RM, May RM. *Infectious diseases of humans: dynamics and control* (Oxford university press) (1991).

[14]Kiss IZ, Miller JC, Simon PL, et al. *Mathematics of epidemics on networks*, *Interdisciplinary Applied Mathematics*, vol. 46 (Springer International Publishing) (2017).

[15]Manica M, Litvinova M, De Bellis A, Guzzetta G, Mancuso P, Vicentini M, et al. Estimation of the incubation period and generation time of sars-cov-2 alpha and delta variants from contact tracing data. *Epidemiology & Infection* **151** (2023) e5. doi:10.1017/S0950268822001947.
